# The corona of a surface bubble promotes electrochemical reactions

**DOI:** 10.1038/s41467-020-20186-0

**Published:** 2020-12-10

**Authors:** Yan B. Vogel, Cameron W. Evans, Mattia Belotti, Longkun Xu, Isabella C. Russell, Li-Juan Yu, Alfred K. K. Fung, Nicholas S. Hill, Nadim Darwish, Vinicius R. Gonçales, Michelle L. Coote, K. Swaminathan Iyer, Simone Ciampi

**Affiliations:** 1 School of Molecular and Life Sciences, Curtin Institute of Functional Molecules and Interfaces, Curtin University, Bentley, WA 6102 Australia; 2 School of Molecular Sciences, The University of Western Australia, Crawley, WA 6009 Australia; 3 ARC Centre of Excellence for Electromaterials Science, Research School of Chemistry, Australian National University, Canberra, ACT 2601 Australia; 4 School of Chemistry, Australian Centre for NanoMedicine and Australian Research Council Centre of Excellence in Convergent Bio-Nano Science and Technology, University of New South Wales, Sydney, NSW 2052 Australia

**Keywords:** Electrocatalysis, Thermodynamics

## Abstract

The evolution of gaseous products is a feature common to several electrochemical processes, often resulting in bubbles adhering to the electrode’s surface. Adherent bubbles reduce the electrode active area, and are therefore generally treated as electrochemically inert entities. Here, we show that this general assumption does not hold for gas bubbles masking anodes operating in water. By means of imaging electrochemiluminescent systems, and by studying the anisotropy of polymer growth around bubbles, we demonstrate that gas cavities adhering to an electrode surface initiate the oxidation of water-soluble species more effectively than electrode areas free of bubbles. The corona of a bubble accumulates hydroxide anions, unbalanced by cations, a phenomenon which causes the oxidation of hydroxide ions to hydroxyl radicals to occur at potentials at least 0.7 V below redox tabled values. The downhill shift of the hydroxide oxidation at the corona of the bubble is likely to be a general mechanism involved in the initiation of heterogeneous electrochemical reactions in water, and could be harnessed in chemical synthesis.

## Abstract (web summary)

Gas bubbles forming on the surface of an electrode, a phenomenon common to several industrial electrolytic processes, are usually perceived as inert, passivating entities. Here, the authors show that that this general assumption does not hold for gas bubbles masking anodes operating in water.

## Introduction

Major industrial processes, such as the electrolysis of alumina^1^, the chloralkali process^2^, and the refining of copper^3^, involve the evolution of gases, which are often accompanied by gas bubbles forming at the electrode. An adherent bubble masks a portion of the electrode; it resists the passage of electrical currents by disrupting ionic conduction at the solid–liquid interface. Surface gaseous cavities are therefore generally regarded as undesirable and electrochemically inactive entities^4^.

The gas–water interface is, however, not electrically neutral^5^, and the interface between water and gas carries electric fields as high as 1.4 V nm^–1^ ^6–8^. Measurements of zeta potential for bubbles suspended in ultrapure water indicate that the corona of a bubble is electrified due to the accumulation of hydroxide ions (OH^−^)^9^. This is most likely caused by the increased self-ionization constant of water at the gas–water interface^10^, which coupled to the fast diffusion of protons by the Grotthuss mechanism^11^, leaves the water surface with an OH^–^ excess. Adhesion of a bubble on a solid surface leads to two interfaces, the solid–gas and the gas–liquid, with the gas obviously acting as an electrically insulating cavity separating the electrode from the liquid^4,12^. However, with an analogy to a suspended gas cavity, at the point where the solid, the gas, and the liquid meet, a high concentration of unbalanced hydroxide ions is likely to exist. We, therefore, postulated that, due to mutual destabilization of hydroxide ions by electrostatic repulsion, the one-electron oxidation of hydroxides to hydroxyls could be significantly facilitated around bubbles adhering to an electrified support. The oxidizing power of hydroxyls (HO^•^ + e^–^ ⇌ OH^–^, E^0^ = +1.90 V vs. SHE)^13^ could then be harvested to trigger redox chemistry^14,15^.

## Results

### Increase in electrochemical current densities in the presence of adherent bubbles

**Fig. 1:**
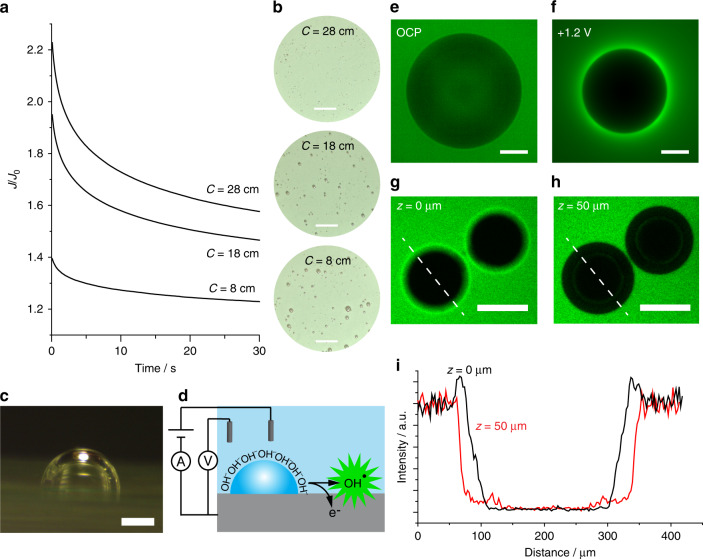
Surface-adherent gas bubbles increase electrochemical current outputs. **a** Normalized amperometric curves acquired in aqueous 0.1 M sodium hydroxide using an ITO electrode, biased at +1.2 V vs. SHE, in the presence of surface-adherent oxygen cavities. The current density recorded in the presence of surface-adherent bubbles (*J*) is systematically higher than that found in the absence of bubbles (*J*_0_). The experimental *J*/*J*_0_ ratio scales with the total bubble corona length (C, data analysis in Supplementary Fig. 2, and Supplementary Table 1). **b** Optical images of the ITO electrode acquired during the current measurements reported in (**a**). Scale bars are 2 mm. Wide-field views of the entire electrode area are in Supplementary Fig. 2. **c** Representative bright-field image (side view) of an oxygen bubble on the ITO electrode. The scale bar is 200 µm. **d** Schematics of the electrochemical generation, and fluorescence detection, of HO^•^ in the corona of an electrode-adherent bubble. **e**, **f** Epifluorescence microscopy images for the detection of HO^•^ around an argon bubble adhering on an ITO electrode. The electrode is immersed in an aqueous solution of sodium hydroxide (0.1 M) and 3ʹ-(p-hydroxyphenyl) fluorescein (10 µM), and it is either rested at its open-circuit potential (**e** OCP), or biased at +1.2 V vs. SHE (**f**). See also Supplementary Video 1. **g**, **h** Confocal microscopy images for the HO^•^ detection around nitrogen bubbles supported on a biased (+1.2 V vs. SHE) ITO slide. The aqueous electrolyte contains dichlorodihydrofluorescein diacetate (100 µM) and sodium hydroxide (0.1 M). The z height above the electrode surface is specified in the figure. Scale bars in **e**–**h** are 200 µm. Emission intensities for the images (frames) in **e**–**h** are normalized to the highest-intensity value measured in each frame. **i** Fluorescence emission intensity profiles measured along the dashed lines of panels **g**, **h**, showing a larger emission at the gas–liquid interface closer to the electrode surface.

Starting with a simple experimental setup, current measurements and optical images in Fig. 1a, b show, unambiguously, that adherent oxygen bubbles (Fig. 1c) are not electrochemically inert entities. In an electrolytic solution containing only 0.1 M sodium hydroxide, the anodic current measured at an indium tin oxide (ITO) electrode, biased at +1.2 V vs. SHE, is surprisingly higher in the presence of macroscopic surface-adherent oxygen bubbles than it is in their absence. An increased electrochemical current is observed in the presence of bubbles, regardless of the gas composition (Supplementary Fig. 1), and the electrochemical current systematically increases by increasing the cumulative bubbles’ perimeter (C, Fig. 1a, b, Supplementary Fig. 2, and Supplementary Table 1). Surface static bubbles favor the oxidation of hydroxides to hydroxyls, and this is a surprising result; a small increase in the gas–water interface outbalances the net loss of “wet” electrode area. We note that these macroscopic bubbles are stable over the time frame of the electrochemical measurements (Supplementary Fig. 3); hence, localized changes in redox reactivity are due to characteristics of the gas–water interface, rather than to energy released during the rapid collapse of the cavity, such as in sonochemistry^16–18^, to convection enhancement^19^, or the cavity absorbing dissolved gas products^20^.

To confirm that the increase in anodic currents, shown in Fig. 1a, is arising from the oxidation of OH^−^ to HO^•^ around adherent gas cavities, we coupled electrochemical experiments to epifluorescence microscopy to visually detect the presence of HO^•^ in the corona of surface bubbles (Fig. 1d). Figure 1e, f (see also Supplementary Video 1) shows epifluorescence microscopy data of argon bubbles resting on an ITO electrode, with 3ʹ-(p-hydroxyphenyl) fluorescein (HPF) present in the electrolytic solution. When the electrode is biased anodically (+1.2 V vs. SHE), a sharp increase in the fluorescence contrast is observed between the bubble’s surface and electrode regions away from the cavity. In our model system, made up of only sodium hydroxide, water, and gas, the plausible reactive oxygen species augmenting the fluorescence of HPF are HO^•^ and H_2_O_2_. HPF is ca. 400 times more selective toward HO^•^ than to H_2_O_2_^21^, strongly suggesting that HO^•^ is being generated in the corona of the bubble. Confocal microscopy images, acquired at different z heights over the electrode surface (Fig. 1g, h), show that hydroxyl radicals are generated in proximity of the gas–water interface, but principally at a height close to the electrode substrate (Fig. 1i).

### Hydroxyl radicals from the oxidation of hydroxide ions

**Fig. 2:**
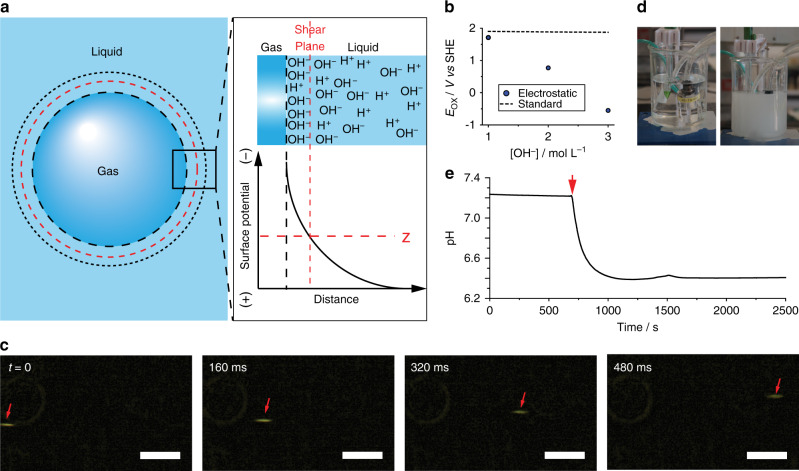
The electric double layer of a bubble in ultrapure water. **a** Simplified schematics of the gas–liquid interface of a bubble, depicting an unbalanced excess of OH^−^ ions. **b** Quantum-chemically computed redox potential for the HO^•^/OH^−^ couple as a function of the OH^−^ concentration, as calculated by considering electrostatic repulsions between OH^−^ (symbols), together with the tabled experimental^13^ potential corresponding to standard conditions of 1 M (dashed line). Details on the calculations are in Supplementary Note 1. **c** Selected time-stamped timeframes obtained from bright-field microscopy imaging experiments (Supplementary Video 2), tracking the position of an oxygen bubble accelerating in ultrapure water toward the anode under an electric field of ca. 40 V cm^−1^. Scale bars are 500 µm. **d** Photographs taken prior (left) and after (right) the formation of a fine gas–water emulsion obtained by generating a high quantity of nitrogen microbubbles (9.3 × 10^7^ L^−1^, average bubble diameter is 50 µm), see Supplementary Note 3. **e** Representative pH drop induced by the formation of a gas emulsion in water.

We note that the increased electrochemical reactivity of surface bubbles observed here is not simply the manifestation of higher current densities observed at regions of an electrode that are partially masked by a dielectric object^4,22^. A local increase in current density implies a reaction rate increase for a favorable reaction, while the oxidation of OH^−^ to HO^•^ is thermodynamically unfavorable at +1.2 V vs SHE. Instead, the contra-thermodynamic shift experienced by the OH^–^ oxidation in the presence of surface-adherent bubbles is more likely linked to the high unbalanced concentration of OH^−^ at the gas–liquid interface (Fig. 2a). Based on Nernst considerations, the HO^•^/OH^–^ redox potential, +1.9 V at standard conditions^13^, will drop only by 59 mV every order of magnitude of increase in OH^–^ activity (Fig. 2b, dashed line). However, if largely unbalanced by cations, electrostatic repulsions between adjacent OH^−^ are likely to further lower their oxidation potential, as demonstrated by our quantum-chemical simulations (Fig. 2b, symbols, and Supplementary Note 1). It was, therefore, necessary to first estimate experimentally the OH^–^ excess concentration at the gas–water interface of bubbles. This excess was first determined by means of accelerating gas bubbles, suspended in ultrapure water, under an electric field (Supplementary Note 2)^9^. Selected video frames (Supplementary Video 2), reproduced in Fig. 2c, show a microscopic (50 µm) oxygen bubble in ultrapure water moving at a velocity of 1.3 mm s^−1^ toward the anode under a field of ca. 40 V cm^−1^. Similar results were obtained for nitrogen bubbles suspended in water (Supplementary Fig. 4 and Supplementary Video 3). The gas–water interface carries an unbalanced population of OH^−^, causing the electrical potential to drop away from the gas–liquid interface (Fig. 2a). A quantitative descriptor of this potential profile is the bubble’s zeta potential *ζ*, that is, the potential difference between the shear plane and the bulk solution^23^. We obtained a *ζ* value of −526 ± 138 mV from the measured bubble velocities using the Smoluchowski equation^24^. The surface charge density of a spherical particle relates to *ζ*, and using the relationship proposed by Loeb et al.^25^, we estimated a charge density of −52 µC cm^−^^2^ or the equivalent of 5.4 × 10^−10^ mol cm^−2^ of OH^−^. To confirm that the negative charge of bubbles is linked to a local unbalanced excess of hydroxide ions, we resorted to direct pH measurements of gas/water emulsions (Fig. 2d). These experiments have strong conceptual analogies with the “pH-stat” experiments reported by Beattie and coworkers for oil emulsions in water^26^. Suspensions of fine nitrogen bubbles are a way to access a water sample characterized by the presence of a very large gas–liquid interface, and as such, a drop in pH would be expected if an excess of OH^−^ is trapped at the interface of water with the gas. By generating a large concentration of microscopic nitrogen bubbles (9.3 × 10^7^ particles L^−1^, Supplementary Video 4) in a water sample, such pH drop was in fact observed (Fig. 2e), indicating at pH ~7 (quiescent sample) a surface OH^−^ excess of 4.2 × 10^−11^ mol cm^−2^ (Supplementary Note 3). At pH ~12, such as where hydroxyl radicals are detected (Fig. 1), this excess rises to 1.3 × 10^−7^ mol cm^−2^ (Supplementary Fig. 5).

### Anisotropic polymerization of luminol around surface bubbles

**Fig. 3:**
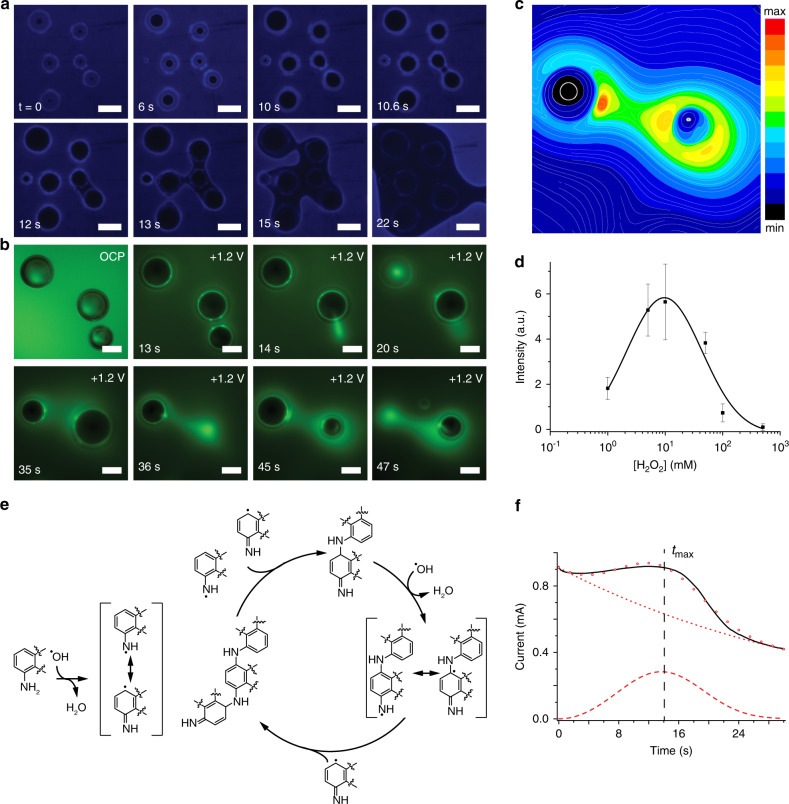
Step polymerization initiated at the corona of surface bubbles. **a** Selected chemiluminescence microscopy (10×, em 440 nm) timeframes (Supplementary Video 5) of the anisotropic polymerization reaction initiated at the gas–liquid interface of oxygen bubbles adhered on an ITO anode (+1.2 V vs. SHE) in an aqueous solution of sodium hydroxide (0.1 M), luminol (0.05 M), and hydrogen peroxide (0.3% v/v). Scale bars are 200 µm. **b** Selected epifluorescence microscopy timeframes (Supplementary Video 7) for the detection of reactive oxygen species around oxygen bubbles adhering on an ITO electrode (OCP and +1.2 V vs. SHE) in a solution containing DCFH_2_-DA (100 µM), sodium hydroxide (0.1 M), and hydrogen peroxide (0.3% v/v). Scale bars are 200 µm. **c** Contour line color map representing the O_2_^•−^ gradient around bubbles, as from the epifluorescence image in (**b**) (*t* = 45 s). **d** Competition of luminol and HO_2_^−^ for HO^•^ revealed by measurements of electrochemiluminescence intensity as a function of the hydrogen peroxide concentration (platinum gauze as working electrode, +1.2 V vs. SHE, 5.0 × 10^−2^ M luminol, 0.1 M sodium hydroxide). Mean values with a 90% confidence limit were calculated based on three independent measurements. The mean values were fitted with a Gaussian function (solid line). **e** Polymerization mechanism. **f** Amperometric curve recorded during the experiment in (**a**), solid line. Symbols are simulated curves, including contributions from a diffusion-limited instantaneous growth (long dashes) and from an electron transfer of an adsorbed electroactive species (short dashes). Details on fitting parameters and the model are in Supplementary Note 7.

To demonstrate the scope and extent to which the surface of an adherent bubble enhances the oxidizing power of an electrode, we designed a detection scheme capable of imaging in real-time the 2D growth of a polymer around bubbles. We coupled an electrochemiluminescent reaction with a step polymerization, both being redox oxidative processes and both being initiated by reactive oxygen species. The surface electrochemiluminescent reaction allows generating a dim internal light source, while the polymer locally quenches it, generating a real-time 2D map of the polymer growth (Fig. 3a, see also Supplementary Videos 5 and 6). We used 5-amino-2,3-dihydrophthalazine-1,4-dione (hereafter luminol) as both the source of chemiluminescence^27,28^ and polymerization reactant^29^. Because the formed polymer is nonconductive in its deprotonated state (Supplementary Note 4), as commonly observed in polyaniline analogs^30^, it impedes electron transfer between the electrode and the electrolyte quenching the electrochemiluminescent reaction.

The anisotropic growth of the polymer filaments (Fig. 3a), linking neighboring bubbles rather than growing radially from the bubble, tracks the concentration gradient of reactive oxygen species away from the bubble. The luminol light path in water begins with the oxidation of the luminol monoanion^31^, yielding a radical susceptible to nucleophilic attack by superoxide (O_2_^•−^). This intermediate collapses to a light emitter excited state of 3-aminophthalate (Supplementary Fig. 6)^15^. Superoxide forms upon the oxidation of hydrogen peroxide by HO^•^ radicals (HO_2_^−^ + HO^•^ → O_2_^•− ^+ H_2_O)^32^, which is evident from the inspection of epifluorescence micrographs obtained around ITO-adherent oxygen (Fig. 3b, and Supplementary Video 7) and argon bubbles (Supplementary Fig. 7, Supplementary Video 8), showing that the presence of oxygen radicals (HO^•^ and O_2_^•−^) at the gas–water interface is far more dramatic in peroxide-containing solutions. We discarded the possibility of homolytic cleavage of HO_2_^–^ to form HO^•^ under the strong electric field at the gas–water interface (Supplementary Note 5). Data in Fig. 3b (and Supplementary Fig. 7) also reveal that the reach of the oxygen radicals away from the bubbles’ surface is dramatically increased when peroxide is present, reflecting the million-fold increase in lifetime for O_2_^•^^−^ over HO^•^^15,33^. The oxygen radical gradient around bubbles is schematized as a color map in Fig. 3c. Bubbles continuously grow as a result of oxygen-evolving upon the oxidation of HO_2_^−^, and they will occasionally collapse or detach (Fig. 3b and Supplementary Video 7).

The chemiluminescence emission is higher at the bubble interface than on the electrode surface, Fig. 3a, as expected from a higher concentration of HO^•^ around bubbles (from OH^−^ oxidation) that react with HO_2_^−^ to generate O_2_^•−^. Luminol can be oxidized to its radical electrochemically (Supplementary Fig. 8), and/or by HO^•^, and O_2_^•−^ forms when HO^•^ oxidizes HO_2_^−^. The latter has to be present in order to observe chemiluminescence. Therefore, both luminol and HO_2_^−^ might compete for HO^•^, which would make the emission intensity reach a maximum value at a specific HO_2_^−^/luminol ratio, and then decrease as this ratio further increases^15^, as was effectively observed (Fig. 3d).

The polymerization reaction starts at the bubble interface from the free oxygen radical attack to luminol (Supplementary Note 6). XPS analysis indicates that multiple pathways are operative, with a film curiously consisting of 50% polyaminophthalate, and 50% of polyaminophthalazine and/or polyluminol (Supplementary Note 4). Theoretical calculations suggest that the polymerization mechanism is a stepwise radical process in which a “step” is first initiated via hydrogen transfer by the HO^•^ radical from aniline functionality of the monomer (Fig. 3e and Supplementary Note 6). Two such resulting radicals couple to form an intermediate that then undergoes a second hydrogen transfer followed by coupling with the radical of a further monomer unit to regenerate the active end group in its nonradical form. In total, three monomer units are joined per step, and four molecules of HO^•^ are consumed. Calculations show that the pathway is feasible for all monomers and hence copolymerization is likely.

Figure 3f (solid line) shows the experimental amperometric curve (electrochemical current vs. time) for the polymerization reaction, with a characteristic shape of an electrodeposition process^34^, confirming that the electrode acts as an electron sink rather than being a homogeneous redox reaction. The electrochemical current goes through a rapid initial increase due to the formation and growth of independent nuclei under hemispherical diffusion control. When these diffusion fronts overlap, as a result of a small separation between nuclei, the overall current reaches a maximum and then starts to decrease. The experimental amperometric curves show a good fitting for a diffusion-limited nucleation growth^34^. The initial exponential decay is due to the oxidation of the adsorbed polymer, and this process was accounted for in the fitting as an additional component over the diffusion-limited instantaneous nucleation, see Supplementary Note 7^35^. The peak maxima observed in Fig. 3f represent the point in time at which diffusion fronts theoretically overlap (*t*_max_), and roughly coincide with the point in Fig. 3a when the polymer films growing between bubbles start overlapping (*t* = 13 s).

Our results demonstrate that at anodes operating in the water, surface static bubbles are not inert cavities but rather highly reactive redox sites. The electrochemical reactivity of an adherent bubble originates from its corona’s ability to accumulate an unbalanced excess of hydroxide anions. In the proximity of the electrode surface, unbalanced anions are oxidized to highly reactive hydroxyl radicals at potentials as low as +1.2 V vs. SHE. The gradient of reactive oxygen species surrounding adherent bubbles can affect anisotropic oxidative redox chemistry. The results presented here are likely to be a general mechanism to initiate, enhance, or localize oxidative processes occurring in water electrolytes.

## Methods

### Chemicals and materials

Unless specified otherwise, all chemicals were of analytical grade and used as received. Hydrogen peroxide (H_2_O_2_, MOS Puranal, 30%, Sigma Aldrich), luminol (97%, Sigma Aldrich), 2′,7′-dichlorodihydrofluorescein diacetate (DCFH_2_-DA, ≥97%, Sigma Aldrich), 3ʹ-(p-hydroxyphenyl) fluorescein (HPF, 5 mM solution in N,N-dimethylformamide, DMF, ThermoFisher), dimethyl sulfoxide (DMSO, 99%, Ajax Finichem), sodium hydroxide (NaOH, 95%, Ajax Finichem), hydrochloric acid (HCl, 37%, Sigma Aldrich), nitric acid (HNO_3_, 70%, Ajax Finichem), sulfuric acid (H_2_SO_4_, 97%, Scharlab), monosodium phosphate (NaH_2_PO_4_, 99%, Sigma Aldrich), oxygen (O_2_, 99.95%, Coregas), nitrogen (N_2_, 99.9%, Coregas), and argon (Ar, 99.997%, Coregas) were used as received. Milli-Q^™^ water (>18 MΩ cm) was used for surface cleaning procedures and for preparing all solutions. ITO-coated glass slides were purchased from Delta Technologies (8–12 Ω/sq in sheet resistance).

### Measurements of the zeta potential of bubbles

To measure the zeta potential of suspended microscopic oxygen and nitrogen bubbles (50 µm in diameter), we used a method based on the procedure of Takahashi^9^. In brief, oxygen or nitrogen gas was bubbled for 1.5 h across an ultrapure water sample held inside a frit-free soda-lime glass H cell (see Supplementary Fig. 9). The cell consisted of two vertical arms of 10 cm in length connected by a horizontal arm 4-cm long. All arms had an internal diameter of 1 cm. A gas dispersion tube of 25–50-µm porosity (Z408743-1EA, Sigma) was used to pass the oxygen or nitrogen gas along with one of the vertical arms. The oxygen or nitrogen flow was stopped, and a bias of 200 V was applied using a Keysight source/measure unit (model B2902A) between two platinum wire electrodes (99.99 + %, 0.5-mm diameter, Goodfellow Cambridge Ltd) inserted in each of the two vertical compartments of the H cell. The bubbles’ movement (speed) in the horizontal channel, and along a horizontal direction pointing from the cathode toward the anode, was used to estimate the bubble zeta potential. Specifically, videos to estimate the bubble position as a function of time were recorded at 25.13 frames per second, using a CCD camera (DCC1240C, Thorlabs) fitted with a 6.5× zoom (MVL6X123Z and MVL133A, Thorlabs). Video recordings were analyzed frame by frame using Fiji image processing package^36^. To minimize electroosmotic forces, possibly interfering with the bubble migration velocity in the field^37^, the focus of the camera was aimed toward the center of the H-cell horizontal arm. This is to minimize surface-related artifacts on the zeta potential measurement. The 95% confidence interval of the zeta potential means is reported as *t*_*n*−1_ *s*/*n*^0.5^, where *t*_*n*−1_ depends on the number of repeats, *s* is the standard deviation, and *n* is the number of independent measurements (which was 11 for the oxygen bubbles and 23 for the nitrogen bubbles)^38^.

### Determination of the OH^−^ excess at the gas–water interface

In addition to quantitative data obtained by electrokinetic experiments (Fig. 2c), the excess of OH^−^ at the interface of a gas bubble suspended in water was also estimated from bulk pH changes (Fig. 2e) recorded when forcing large fluxes of microscopic nitrogen (99.9%) bubbles in a water sample (Fig. 2d). These vigorously aerated samples have a large gas–water interface area (surface-to-volume ratio, determined by optical microscopy) and were obtained by flowing an aqueous solution (800 mL in a 1-L borosilicate glass beaker) of potassium chloride (0.1 M) of variable pH (adjusted by dropwise addition of a sodium hydroxide aqueous solution (0.1 M)) through a microbubble generator nozzle (CARMIN D2, Ylec, France). The solution was first deaerated for 30 min by flowing nitrogen gas through the solution using a gas dispersion tube of 25–50-µm porosity (Z408743-1EA, Sigma), while water was pumped (and continuously recirculated) by a diaphragm pump (Xylem Flojet AD49/0) at a liquid flow of 3 L/min. The gas dispersion tube was disconnected and the pH allowed to stabilize over a time frame >600 s (dpH/d*t* < 0.002 units min^−1^, Fig. 2e and Supplementary Fig. 5). The nozzle was then connected to the pump circuit. The design of the aerator is that the flowing liquid causes concomitant suction of a gas, in this case, ultra-high-purity nitrogen gas (99.9%, Coregas), into the solution, through the nozzle. A low-conductivity pH probe (model HI1053, Hanna Instruments), connected to a pH meter (HI5221) with computer connectivity, was used to monitor the solution pH over time. All measurements were performed inside an acrylate glovebox kept under positive nitrogen pressure (99.9%, Coregas). Videos, used to estimate the total surface-to-volume ratio of the suspended bubbles, were recorded using a CCD camera (DCC1240C, Thorlabs) fitted with a 6.5× zoom. The experimental setup is shown in Supplementary Fig. 10.

### Deposition of bubbles on the electrode

Oxygen, nitrogen, and argon bubbles were deposited on the ITO-coated glass electrode with the aid of a gas dispersion tube of 10–20-µm porosity (Z408727, Sigma Aldrich). In brief, the electrochemical cell holding the ITO electrode was initially filled with water, then the gas flow was forced across the liquid, and after having visually inspected the ITO slide to confirm the presence of adherent gas bubbles, a concentrated solution containing the chemical species of interest was added so to reach a specific final concentration.

### Amperometry, epifluorescence, and electrochemiluminescence microscopy

A custom-made three-electrode single-compartment electrochemical cell (Supplementary Fig. 11) was used for all electrochemical and fluorescence experiments. An ITO-coated glass slide served as the working electrode (7.1 cm^2^, geometric area), a platinum foil as the counter electrode (25.8 cm^2^, geometric area), and an Ag|AgCl|KCl (sat.) electrode as reference. The cell’s ohmic resistance was 13 ohm, measured in a 0.1 M aqueous solution of sodium hydroxide. The counter- and reference electrodes are kept at a distance of 1 cm from the working electrode, and the cell was generally loaded with 20 mL of electrolyte/fluorophore solutions. The three electrodes were connected to an EmStat3 potentiostat (PalmSens BV). All potentials are reported against the standard hydrogen electrode (SHE). Luminol electrochemiluminescence and fluorescein fluorescence were detected using a Nikon Eclipse Ti2 inverted fluorescence microscope equipped with a 14-bit monochromatic camera (Nikon DS-Qi2), Plan Fluor 10×/0.30 Ph1 objective, and Semrock quad-band excitation/emission filter (LED-DA/FI/TR/Cy5). Images were captured at 1024 × 1024-pixel (px) resolution. For the detection of OH^•^ and OH^−^, 470-nm excitation and 515-nm emission were used. For the detection of luminol electrochemiluminescence, the sample was kept in the dark and no emission filter was used. For the wide-field videos (Supplementary Video 6), the luminol electrochemiluminescence was recorded using a CMOS camera (CS235CU, Thorlabs) equipped with a focusing lens (MVL50M23, Thorlabs, 50 mm, f/2.8). For the detection of reactive oxygen species by fluorescence, we used aqueous solutions containing either 1.0 × 10^−4^ M DCFH_2_-DA or 1.0 × 10^−5^ M HPF. The 1.0 × 10^−4^ M DCFH_2_-DA solution was prepared from a 1.0 × 10^−3^ M DCFH_2_-DA stock solution in dimethyl sulfoxide so that the final concentration of dimethyl sulfoxide during the imaging experiments is 1% v/v. The 1.0 × 10^−5^ M HPF solution was prepared from a 5.0 × 10^−3^ M DCFH_2_-DA stock solution in DMF, with a 0.4% v/v final concentration of DMF. For the electrochemiluminescence detection, an aqueous solution of 0.1 M sodium hydroxide, 0.05 M luminol, and 0.3% v/v hydrogen peroxide was used.

### Confocal microscopy

12-bit fluorescence and differential interference contrast microscopy images at 512 × 512-px resolution were captured using a Nikon A1R laser scanning confocal system attached to a Nikon Ti-E inverted microscope, using a Plan Apo λ 10×/0.45 objective with excitation set to 488 nm, emission to 525/50 nm, and with a DU4 detector. Images were captured at 1.00× zoom and a pinhole size of 17.9 µm. The electrochemical cell was the same as that used for the epifluorescence and chemiluminescence microscopy experiments.

### Competition of H_2_O_2_ and luminol for HO^•^

The competition of hydrogen peroxide and luminol for HO^•^ as their oxidant was studied by electrochemiluminescence spectroscopy with a Cary Eclipse Fluorescence spectrophotometer, using a spectroelectrochemical cell from BASi (EF-1362) fitted with a platinum gauze as the working electrode, a platinum wire as the counter electrode, and an Ag/AgCl/KCl (sat.) as the reference electrode. All electrochemical measurements were performed using a potentiostat from PalmSens BV (EmStat3). All potentials are reported against the SHE. The electrochemiluminescence intensity was measured at the peak maxima (425 nm) at an applied potential of +1.2 V in a solution containing 5.0 × 10^−2^ M luminol, 0.1 M sodium hydroxide, and concentrations of hydrogen peroxide ranging from 1 × 10^−3^ to 0.5 M. The platinum electrodes were cleaned after each measurement by electrochemical cycling (20 cycles at a sweep rate of 0.1 V/s) in 0.5 M aqueous nitric acid and then rinsed with Milli-Q^™^ water.

### X-ray photoelectron spectroscopy

X-ray photoelectron spectroscopy was performed with an ESCALab 250 Xi spectrometer (ThermoFisher Scientific) fitted with a monochromated Al Kα source. The pressure in the analysis chamber during measurements was <10^−8^ mbar. The pass energy and step size for narrow scans were 20 and 0.1 eV, respectively, and the take-off angle was normal to the sample surface. Spectral analysis was performed by using the Avantage 4.73 software and curve fittings were carried out with a mixture of Gaussian–Lorentzian functions. Emission peaks were calibrated by applying a rigid binding energy shift to bring the C1s emission of the C − C signal to 284.3 eV.

### Theoretical procedures

Quantum-chemical calculations were undertaken to assess the possibility of electrostatically driven peroxide homolysis, to determine the effect of charge repulsion on hydroxide oxidation, and to ascertain the mechanism of luminol polymerization. A summary of the methods is given below, further details are provided in the relevant Supplementary Notes.

Calculations on peroxide homolysis and luminol polymerization were carried out with the Gaussian 16.C01^39^ software package, at the M06-2X/6-31 + G(d,p) level of theory for both geometry optimizations and frequency calculations. Where relevant, conformational searching was also carried out at this level using the energy-directed tree search algorithm^40^. For peroxide homolysis, a thermocycle approach was used to obtain aqueous-phase Gibbs free energies from gas-phase Gibbs free energies and SMD^41^ solvation energies. For the polymerization, geometries in the gas and aqueous phases were significantly different, and so the direct method^42^ was used instead. In both cases, standard partition functions for an ideal gas under the harmonic oscillator-rigid rotor approximation were used. For peroxide homolysis, the electric field was applied using the field command in Gaussian with values of 2 and 10 atomic units, the former corresponding best to the estimated field at the bubble surface^6–8^. Multiple directions were screened and the most stabilizing directions applied in all cases.

To assess the effects of charge repulsion on hydroxide oxidation, a 1 M solvent system comprising 1 hydroxide and 52 water molecules was extracted from the trajectory of 1 ns NVT simulation of a system containing 1 hydroxide and 100 water molecules in cubic boxes with a length of 14.46 angstrom based on the density of water. The initial cubic box was set up using the Packmol program^43^, and the Travis program^44^ was used to place a solute molecule (HO^−^ or HO^•^) in the center of each simulation box. For each cluster, the GFN2-xTB method^45^ implemented in the xtb^46^ code (version 6.2.3) is used to optimize the structure, and the most stable cluster is taken for further optimization using the B97-3c method^47^ with the ORCA program^48^. Improved energies were calculated with RI-PWPB95-D3(BJ)^49,50^/def2-QZVPP^51^ single-point energies and these were further corrected to the CCSD(T)/CBS^52,53^ level via an ONIOM approximation^54^ in which the core system was the isolated reagent. Oxidation potentials were computed using a value of 4.281V^55^ for the reference electrode and Boltzmann statistics for the electron. The effect of electrostatic repulsion was assessed by repeating the calculations for simulation boxes in which 1 (2 M) or 2 (3 M) extra HO^−^ ions were included.

## Supplementary information

Supplementary Information

Description of Additional Supplementary Files

Video 1

Video 2

Video 3

Video 4

Video 5

Video 6

Video 7

Video 8

## Data Availability

Data supporting the findings of this work are available within the paper and its Supplementary Information files. The optimized coordinates for the OH^−^ and HO^•^ systems are deposited at https://figshare.com/s/a547a7a21d4a2d37ba73.
