# Supplementary material for: The corona of a surface bubble promotes electrochemical reactions
Source: Nat Commun. 2020 Dec 10;11:6323. doi: 10.1038/s41467-020-20186-0 (PMC7729901; doi:10.1038/s41467-020-20186-0)
Supplement: Supplementary file 1 — Supplementary Information [file 41467_2020_20186_MOESM1_ESM.pdf]

Supplementary Information for

The Corona of a Surface Bubble Promotes Electrochemical Reactions

Yan B. Vogel,[1] Cameron W. Evans,[2] Mattia Belotti,[1] Longkun Xu,[3] Isabella C. Russell,[3] Li-Juan Yu,[3] Alfred K.K. Fung,[3] Nicholas S. Hill,[3] Nadim Darwish,[1] Vinicius R. Gonçales,[4] Michelle L. Coote,[3] K. Swaminathan Iyer[2] & Simone Ciampi[1]

[1]School of Molecular and Life Sciences, Curtin Institute of Functional Molecules and Interfaces, Curtin University, Bentley, Western Australia 6102, Australia

[2]School of Molecular Sciences, The University of Western Australia, Crawley, Western Australia 6009, Australia

[3]ARC Centre of Excellence for Electromaterials Science, Research School of Chemistry, Australian National University, Canberra, Australian Capital Territory 2601, Australia

[4]School of Chemistry, Australian Centre for NanoMedicine and Australian Research Council Centre of Excellence in Convergent Bio-Nano Science and Technology, University of New South Wales, Sydney, New South Wales 2052, Australia

Table of Contents

Supplementary Fig. 1. Electrochemical reactivity of nitrogen and argon bubbles ……….... 3

Supplementary Fig. 2. Optical images corresponding to the data in Fig. 1a of the main text ……………………………………………………………………………………4

Supplementary Fig. 3. Stability of surface-adherent bubbles ……………………………… 5

Supplementary Fig. 4. Electrokinetic experiments with nitrogen bubbles ………………… 6

Supplementary Fig. 5. pH drop induced by a massive increase of the air–water interface ... 7

Supplementary Fig. 6. Mechanism of luminol electrochemiluminescence ……………….... 8

Supplementary Fig. 7. Hydroxyl radicals generated preferentially around surface argon bubbles ……………………………………………………………………………… 9

Supplementary Fig. 8. Electrochemical oxidation/reduction of luminol …………………. 10

Supplementary Fig. 9. Experimental setup for the measurement of the zeta potential of suspended bubbles ……………………………………………………………………….. 11

Supplementary Fig. 10. Experimental setup for microbubble generation ………………... 12

Supplementary Fig. 11. Custom-made three-electrode electrochemical cell for the electrochemical and microscopy experiments ……………………………………………… 13

Supplementary Table 1. Analysis of the total bubble coverage area and circumference length of Supplementary Fig. 2 ……………………………………………………….. 14

Supplementary Note 1: Electrostatic repulsions lower the oxidation potential of hydroxide anions ……………………………………………………………………………… 20

Supplementary Note 2: Determination of the hydroxide ion concentration surrounding a bubble 23

Supplementary Note 3: presence of an OH– excess at the gas–water interface, as determined by bulk pH measurements in gas/water emulsions 24

Supplementary Note 4: Electrodeposited film characterization 25

Supplementary Note 5: HO• cannot form from HOO– bond cleavage 26

Supplementary Note 6: Polymerization mechanism 28

Supplementary Note 7: Electrodeposition curve fitting 33

Supplementary References 34

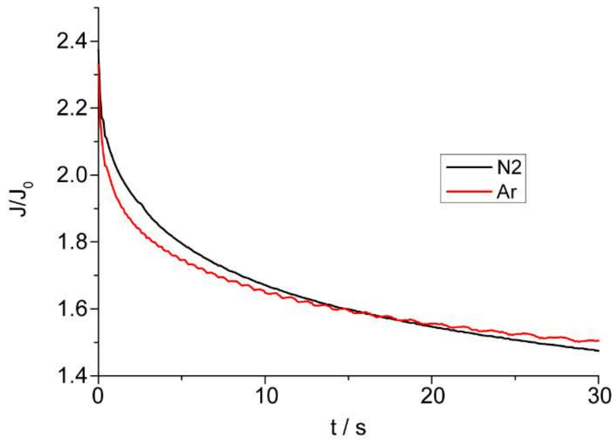

Supplementary Fig. 1. Electrochemical reactivity of nitrogen and argon bubbles. Normalized amperometric curves for an ITO electrode biased at +1.2 V *vs* SHE in the presence of surface-adherent nitrogen and argon bubbles in an aqueous 0.1 M sodium hydroxide solution. The current density in the presence of surface adherent bubbles (J) is systematically higher than in the absence of bubbles (J_0). The total circumference of adherent bubbles is 27 cm (N_2), and 32 (Ar).

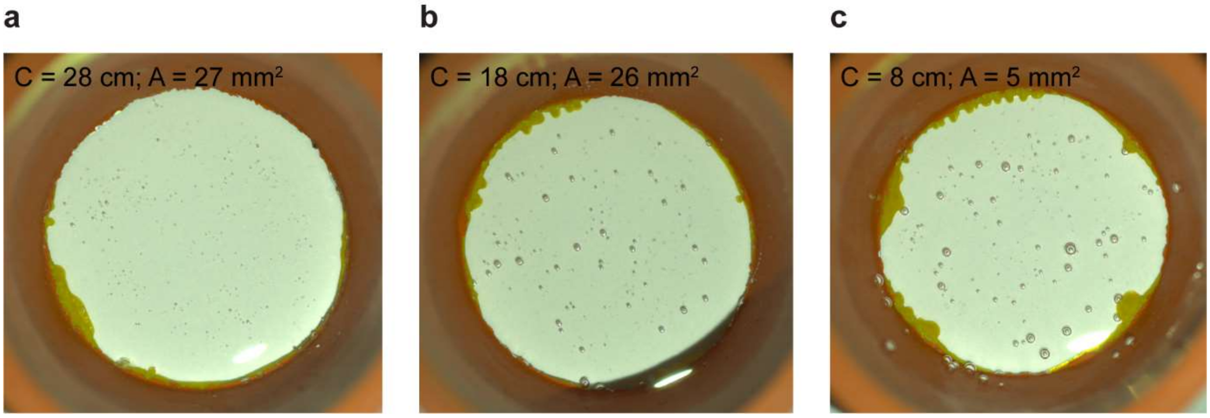

Supplementary Fig. 2. Optical images corresponding to the data in Fig. 1a of the main text. The total bubble circumference (C) and total area covered by bubbles (A) are indicated by the labels found in the upper left corner of each image. Comprehensive data on sizes and amount of bubbles for each image are listed in Supplementary Table 1. The electrode diameter is 3 cm.

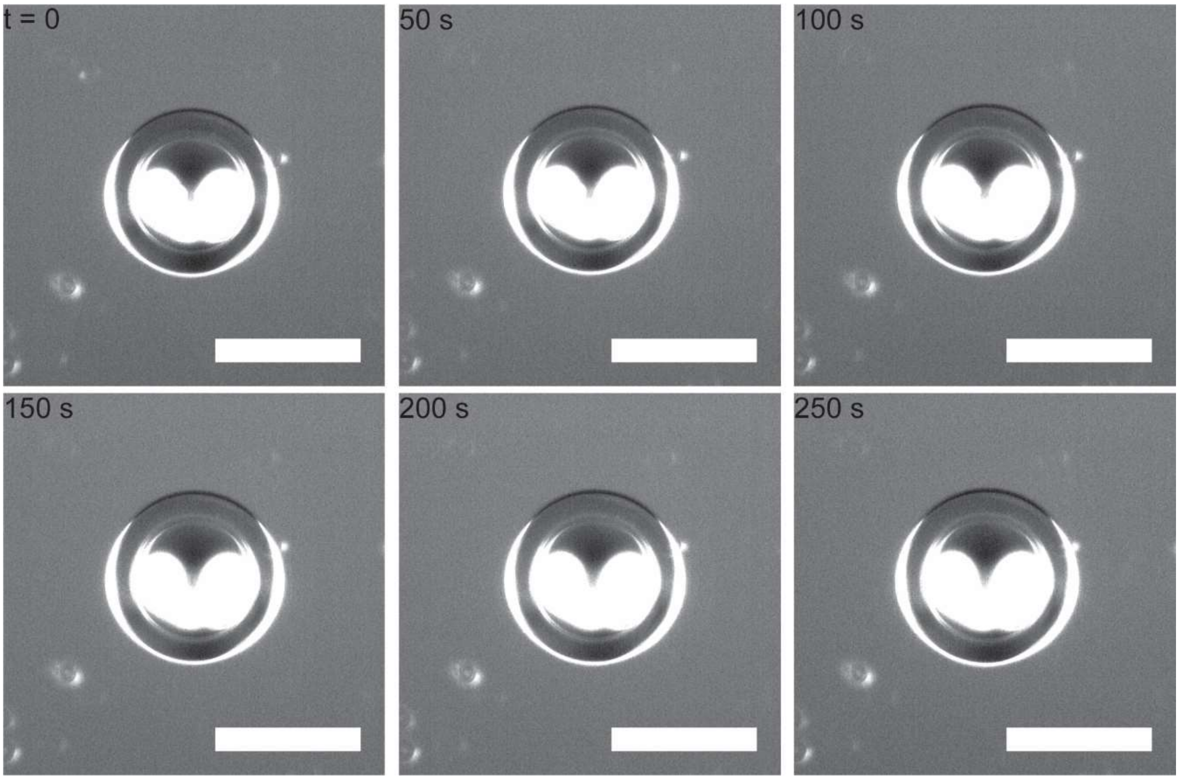

Supplementary Fig. 3. Stability of surface-adherent bubbles. Bright field optical microscopy images of an argon bubble adhering onto an ITO electrode under a bias of +1.2 V *vs* SHE. The electrolyte is aqueous sodium hydroxide (0.1 M). The time label shown in the top left corner of each panel specifies the time lapsed after the potential step was applied. Scale bars are 200 μm.

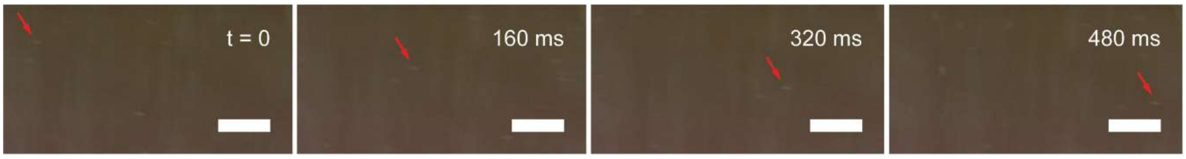

Supplementary Fig. 4. Electrokinetic experiments with nitrogen bubbles. Selected time-stamped frames (time is shown on the up-right corner), part of a bright field microscopy recording, tracking the position of a nitrogen bubble accelerating towards the anode under an electric field of 40 V cm^{-1} in ultrapure water. The zeta potential was obtained from the analysis of the initial velocity of 23 independent measurements, and is 644 ± 124 mV. Scale bars are 500 µm.

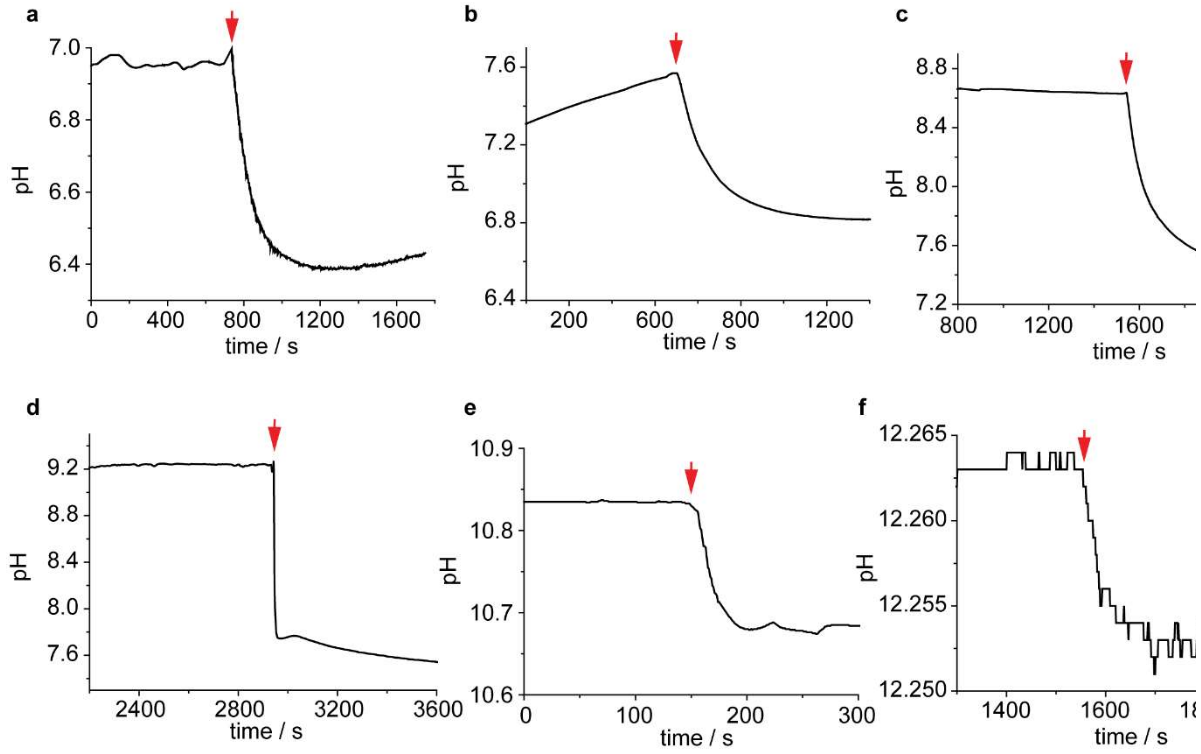

Supplementary Fig. 5. pH drop induced by a massive increase of the air-water interface. a–f Representative data indicating a bulk pH drop measured upon the formation of a gas/water emulsion with inherently high interfacial area (ca. 3×10^{3} cm^{2}, as measured by optical microscopy, Supplementary Video 4) This bulk measurement, performed as a function of the pH of the quiescent sample, allows to determine the surface OH^{-} concentration (at the newly formed gas–water interface), which was 2.4×10^{-11} mol cm^{-2} (**a**), 1.1×10^{-10} mol cm^{-2} (**b**), 1.3×10^{-9} mol cm^{-2} (**c**), 5.5×10^{-9} mol cm^{-2} (**d**), 6.8×10^{-8} mol cm^{-2} (**e**), and 1.3×10^{-7} mol cm^{-2} (**f**). The pH was adjusted by adding dropwise a sodium hydroxide solution (0.1 M) into an aqueous solution of potassium chloride (0.1 M). This solution was allowed to equilibrate under nitrogen gas until the pH stabilised (dpH/dt <0.002 units min^{-1}), refer to the methods section in the main text for details. At this point a flow of nitrogen microbubbles was forced through the sample (arrow symbols in figure). Refer to the methods section in the main text for further experimental details.

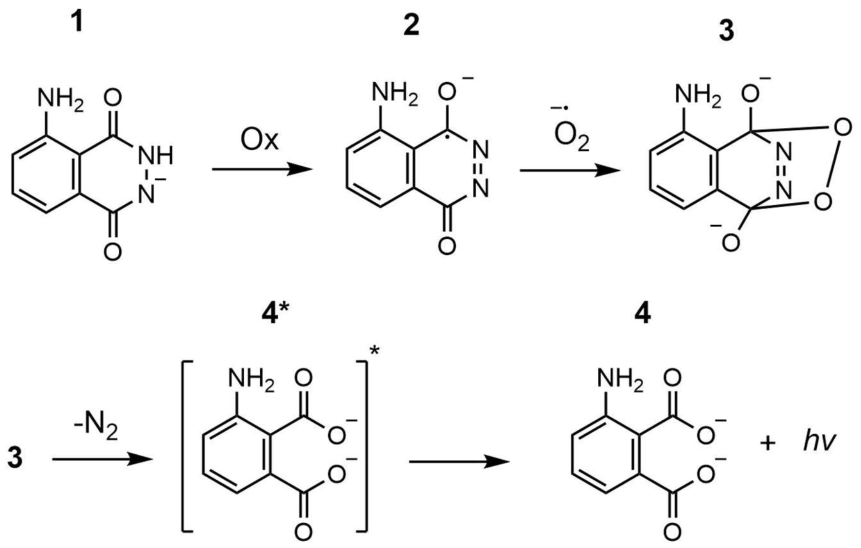

Supplementary Fig. 6. Mechanism of luminol electrochemiluminescence. At the pH studied here, luminol exists as its monoanion (**1**).[1] When a potential of +1.2 V *vs* SHE is applied to a metallic electrode, **1** is oxidized (see Supplementary Fig. 8) to its radical (**2**). The luminol monoanion can also be oxidized by $HO^{\bullet}$ (E^0 = 1.9 V *vs* SHE).[2] Then **2** is attacked by $O_2^{\bullet -}$ to form an endoperoxide intermediate (**3**) that leads to an excited state of 3-aminophtalate (**4***) which is the light-emitter and radiatively decays to **4**.[3]

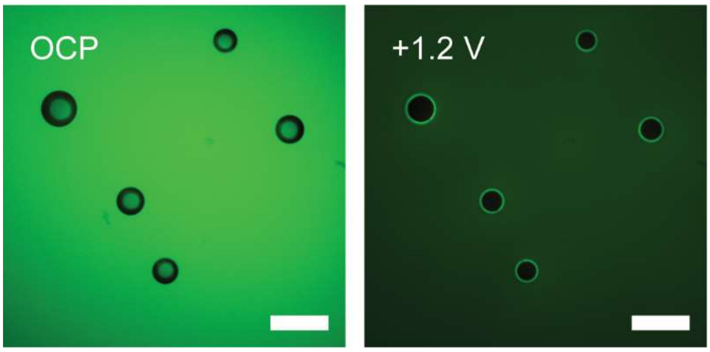

Supplementary Fig. 7. Hydroxyl radicals generated preferentially in the corona of surface-adherent argon bubbles. Epifluorescence microscopy images of argon bubbles deposited on an ITO-coated glass slide in an argon-saturated aqueous solution containing 0.1 M phosphate buffer at pH 7, 100 µM DCFH$_2$-DA and 0.3% H_2O_2, before (i.e. at OCP) and after applying an electrode potential of +1.2 V. Scale bars are 200 µm.

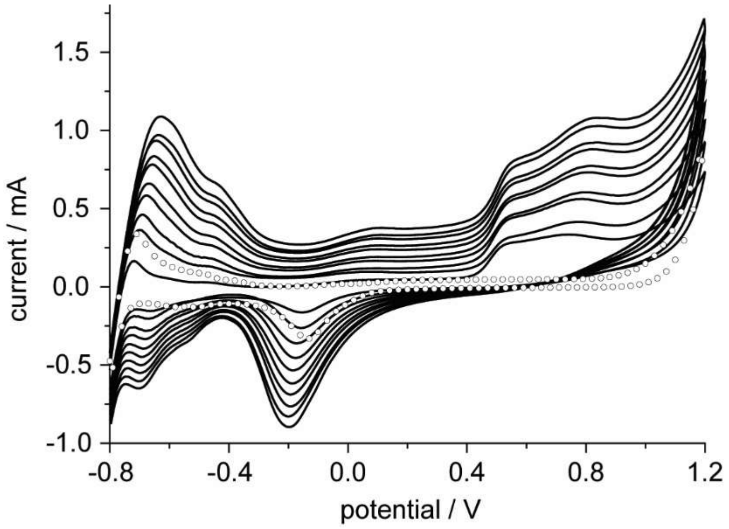

Supplementary Fig. 8. Electrochemical oxidation/reduction of luminol. Cyclic voltammetry of luminol (5.0×10^{-3} M) in 0.1 M sodium hydroxide using a platinum wire as the working electrode (solid black lines). The voltage scan rate was varied from 0.1 V/s to 1 V/s, in increments of 0.1 V/s. The dotted trace indicates a cyclic voltammogram (0.1 V/s) acquired in 0.1 M sodium hydroxide only.

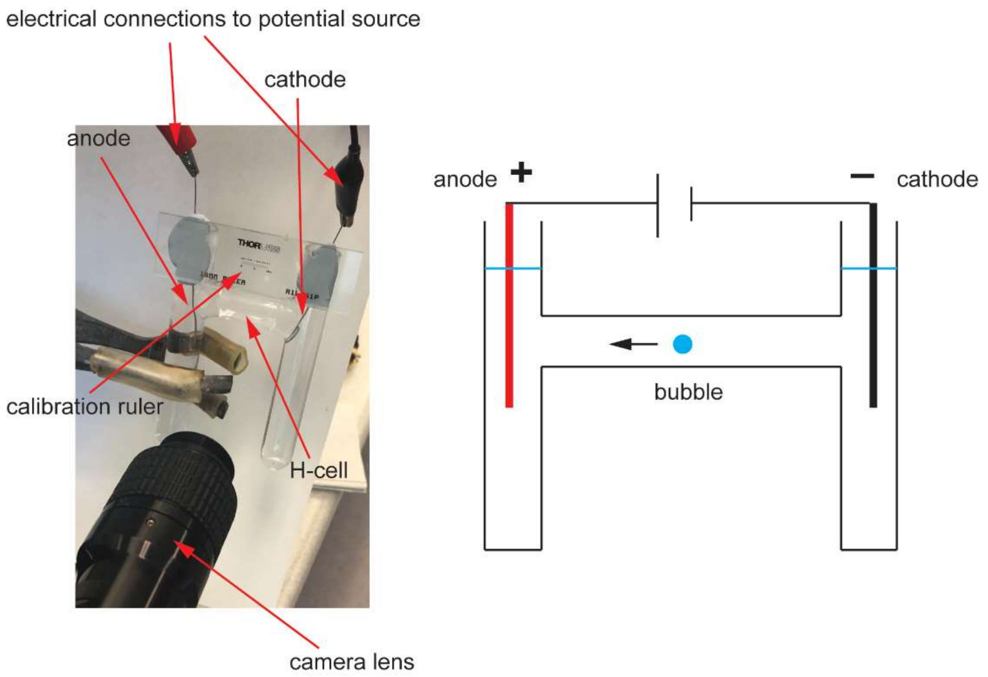

Supplementary Fig. 9. Experimental setup for the measurement of the zeta potential of suspended bubbles. Picture (left) and schematics (right) of the experimental setup used to accelerate, and track the movement, of microscopic oxygen or nitrogen bubbles in water under a d.c. electric field (ca. 40 V/cm). Refer to the Methods section in the main text for details on the measurement.

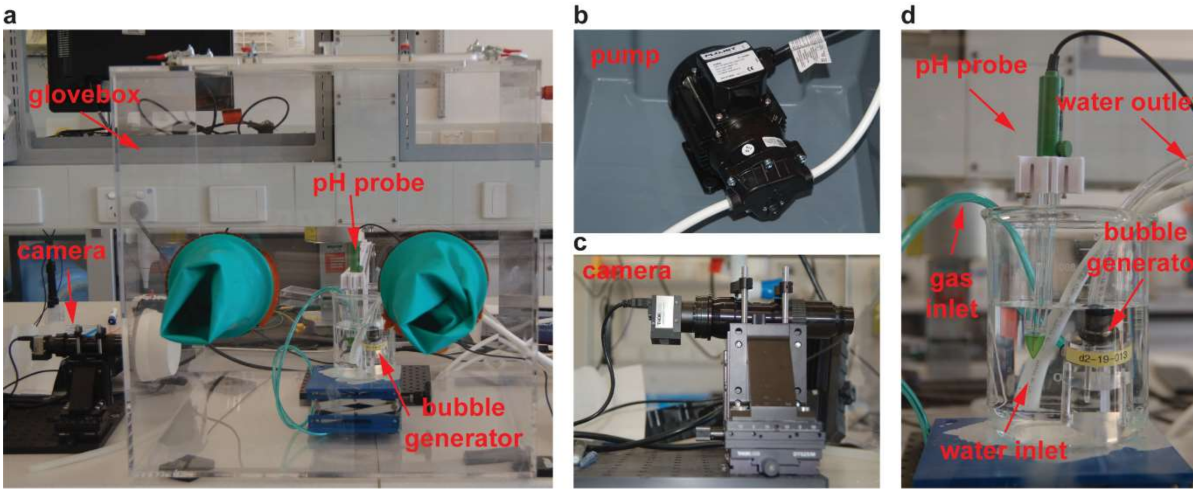

Supplementary Fig. 10. Experimental setup for microbubble generation. Photographs showing the experimental setup used for the determination of the OH^- excess at the gas–water interface. **a** Inert atmosphere glovebox. **b** Water pump (Xylem Flojet AD4/90). **c** High magnification CCD camera (DCC1240C, Thorlabs) fitted with a 6.5× zoom (MVL6X123Z and MVL133A, Thorlabs). **d** Low conductivity samples pH probe, and microbubble generator nozzle (Carmin D2, Ylec, production of over 10^6 bubbles/s).

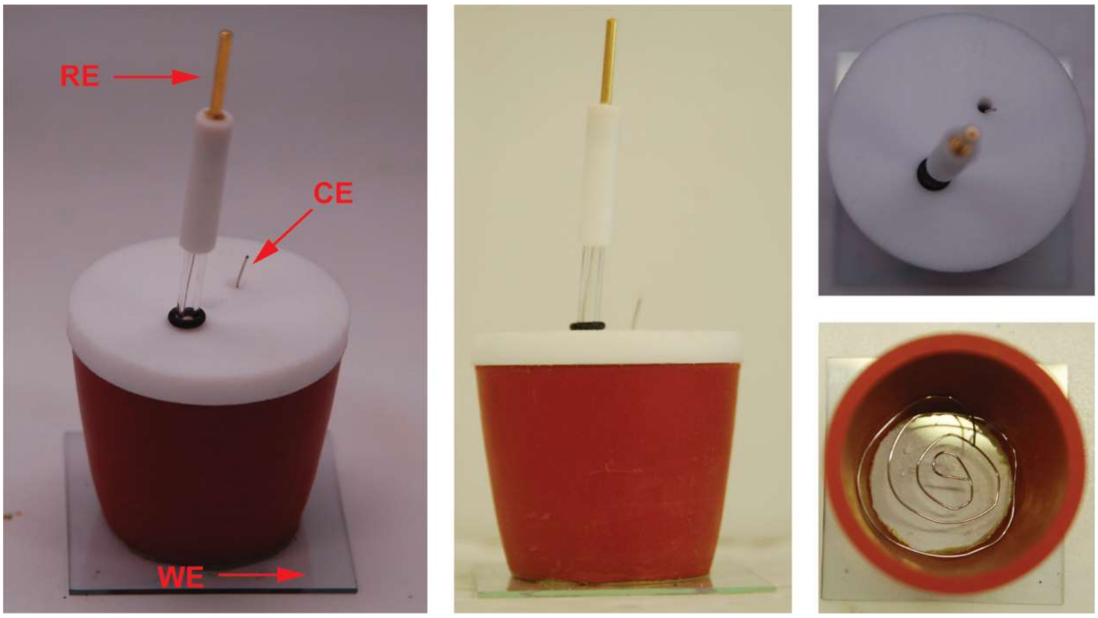

Supplementary Fig. 11. **Custom-made three-electrode electrochemical cell for the electrochemical and microscopy experiments.** Images of the three-electrode and single-compartment electrochemical cell used for electrochemical and microscopy experiments. The reference electrode (RE label in figure) is an Ag|AgCl|KCl (sat.) electrode, the counter (CE) is a platinum coil that covers the entire area of the undelaying ITO slide (WE) resting at a fix vertical distance from it (10 mm). A hydrocarbon-based glue was used to secure a rubber filter adapter cone to the WE surface, so to contain electrolyte/fluorophore solutions.

Supplementary Table 1. Analysis of the total bubble coverage area and circumference length of Supplementary Fig. 2

	a	a	a	b	b	b	c	c	c
n	area	radius	circumference	area	radius	circumference	area	radius	circumference
	mm	mm		mm	mm		mm	mm	
1	0.015	0.0691	0.43416	9.63E-04	0.0175	0.10998	0.055	0.13231	0.83135
2	0.021	0.08176	0.51371	1.843	0.76593	4.81247	0.006	0.0437	0.27459
3	0.209	0.25793	1.62061	0.006	0.0437	0.27459	9.63E-04	0.0175	0.10998
4	0.011	0.05917	0.37179	0.586	0.43189	2.71365	9.63E-04	0.0175	0.10998
5	0.253	0.28378	1.78306	1.706	0.73691	4.63014	9.63E-04	0.0175	0.10998
6	0.007	0.0472	0.29659	0.016	0.07136	0.4484	0.003	0.0309	0.19416
7	0.371	0.34365	2.1592	0.235	0.2735	1.71846	0.119	0.19462	1.22286
8	9.63E-04	0.0175	0.10998	0.816	0.50965	3.20221	0.008	0.05046	0.31707
9	0.054	0.13111	0.82376	9.63E-04	0.0175	0.10998	0.002	0.02523	0.15853
10	9.63E-04	0.0175	0.10998	0.803	0.50557	3.1766	0.082	0.16156	1.01511
11	0.365	0.34086	2.14166	0.149	0.21778	1.36835	0.078	0.15757	0.99004
12	9.63E-04	0.0175	0.10998	9.63E-04	0.0175	0.10998	0.408	0.36038	2.26431
13	0.013	0.06433	0.40418	9.63E-04	0.0175	0.10998	0.002	0.02523	0.15853
14	0.306	0.31209	1.96095	0.142	0.2126	1.33582	9.63E-04	0.0175	0.10998
15	0.152	0.21996	1.38206	0.14	0.2111	1.32638	0.003	0.0309	0.19416
16	0.009	0.05352	0.3363	0.018	0.07569	0.4756	0.002	0.02523	0.15853
17	0.108	0.18541	1.16498	0.379	0.34733	2.18235	0.015	0.0691	0.43416
18	9.63E-04	0.0175	0.10998	0.019	0.07777	0.48863	0.002	0.02523	0.15853
19	0.019	0.07777	0.48863	0.081	0.16057	1.0089	0.002	0.02523	0.15853
20	0.167	0.23056	1.44865	0.053	0.12989	0.8161	9.63E-04	0.0175	0.10998
21	0.082	0.16156	1.01511	0.305	0.31158	1.95774	0.047	0.12231	0.76852
22	0.004	0.03568	0.2242	1.264	0.63431	3.98546	9.63E-04	0.0175	0.10998
23	9.63E-04	0.0175	0.10998	9.63E-04	0.0175	0.10998	0.004	0.03568	0.2242
24	0.043	0.11699	0.73509	0.119	0.19462	1.22286	0.06	0.1382	0.86832
25	0.011	0.05917	0.37179	0.002	0.02523	0.15853	0.338	0.32801	2.06093
26	0.013	0.06433	0.40418	0.316	0.31715	1.99273	9.63E-04	0.0175	0.10998
27	0.012	0.0618	0.38833	0.002	0.02523	0.15853	0.004	0.03568	0.2242
28	0.015	0.0691	0.43416	0.002	0.02523	0.15853	0.002	0.02523	0.15853
29	9.63E-04	0.0175	0.10998	9.63E-04	0.0175	0.10998	0.002	0.02523	0.15853
30	0.003	0.0309	0.19416	9.63E-04	0.0175	0.10998	9.63E-04	0.0175	0.10998
31	9.63E-04	0.0175	0.10998	9.63E-04	0.0175	0.10998	0.004	0.03568	0.2242
32	9.63E-04	0.0175	0.10998	9.63E-04	0.0175	0.10998	0.337	0.32752	2.05788
33	0.008	0.05046	0.31707	9.63E-04	0.0175	0.10998	0.003	0.0309	0.19416
34	0.336	0.32704	2.05482	9.63E-04	0.0175	0.10998	0.01	0.05642	0.35449
35	0.683	0.46627	2.92965	9.63E-04	0.0175	0.10998	9.63E-04	0.0175	0.10998
36	0.093	0.17205	1.08105	9.63E-04	0.0175	0.10998	0.002	0.02523	0.15853
37	0.329	0.32361	2.03331	9.63E-04	0.0175	0.10998	9.63E-04	0.0175	0.10998
38	0.5	0.39894	2.50663	0.009	0.05352	0.3363	0.003	0.0309	0.19416
39	0.094	0.17298	1.08685	0.849	0.51985	3.26632	0.003	0.0309	0.19416
40	0.119	0.19462	1.22286	9.63E-04	0.0175	0.10998	9.63E-04	0.0175	0.10998
41	0.089	0.16831	1.05755	0.004	0.03568	0.2242	0.098	0.17662	1.10973
42	0.084	0.16352	1.02741	0.003	0.0309	0.19416	9.63E-04	0.0175	0.10998
43	0.072	0.15139	0.9512	0.084	0.16352	1.02741	0.008	0.05046	0.31707
44	0.39	0.35234	2.21379	0.022	0.08368	0.52579	0.03	0.09772	0.614
45	0.044	0.11835	0.74359	0.004	0.03568	0.2242	0.005	0.03989	0.25066
46	0.079	0.15858	0.99637	0.02	0.07979	0.50133	9.63E-04	0.0175	0.10998
47	0.116	0.19216	1.20735	0.013	0.06433	0.40418	9.63E-04	0.0175	0.10998
48	0.073	0.15244	0.95778	9.63E-04	0.0175	0.10998	9.63E-04	0.0175	0.10998
49	0.109	0.18627	1.17036	0.003	0.0309	0.19416	0.002	0.02523	0.15853
50	0.195	0.24914	1.56539	0.012	0.0618	0.38833	0.097	0.17572	1.10406
51	9.63E-04	0.0175	0.10998	9.63E-04	0.0175	0.10998	9.63E-04	0.0175	0.10998
52	0.058	0.13587	0.85373	0.013	0.06433	0.40418	0.01	0.05642	0.35449
53	0.054	0.13111	0.82376	0.014	0.06676	0.41944	9.63E-04	0.0175	0.10998
54	0.04	0.11284	0.70898	0.011	0.05917	0.37179	0.003	0.0309	0.19416
55	9.63E-04	0.0175	0.10998	0.014	0.06676	0.41944	9.63E-04	0.0175	0.10998
56	0.352	0.33473	2.10318	0.008	0.05046	0.31707	9.63E-04	0.0175	0.10998
57	0.085	0.16449	1.03351	0.009	0.05352	0.3363	0.005	0.03989	0.25066
58	0.081	0.16057	1.0089	0.006	0.0437	0.27459	0.007	0.0472	0.29659
59	0.343	0.33042	2.07612	0.013	0.06433	0.40418	0.039	0.11142	0.70006

60	0.266	0.29098	1.82829	0.006	0.0437	0.27459	9.63E-04	0.0175	0.10998
61	0.622	0.44496	2.79576	0.347	0.33235	2.08819	0.002	0.02523	0.15853
62	9.63E-04	0.0175	0.10998	0.137	0.20883	1.31209	9.63E-04	0.0175	0.10998
63	9.63E-04	0.0175	0.10998	9.63E-04	0.0175	0.10998	9.63E-04	0.0175	0.10998
64	0.013	0.06433	0.40418	0.002	0.02523	0.15853	9.63E-04	0.0175	0.10998
65	0.055	0.13231	0.83135	0.011	0.05917	0.37179	0.003	0.0309	0.19416
66	0.01	0.05642	0.35449	9.63E-04	0.0175	0.10998	0.003	0.0309	0.19416
67	0.051	0.12741	0.80055	0.302	0.31005	1.94809	0.003	0.0309	0.19416
68	0.054	0.13111	0.82376	0.002	0.02523	0.15853	0.028	0.09441	0.59318
69	0.135	0.2073	1.30248	0.02	0.07979	0.50133	9.63E-04	0.0175	0.10998
70	0.004	0.03568	0.2242	0.221	0.26523	1.66648	9.63E-04	0.0175	0.10998
71	0.161	0.22638	1.42239	0.379	0.34733	2.18235	9.63E-04	0.0175	0.10998
72	0.077	0.15656	0.98367	0.011	0.05917	0.37179	0.006	0.0437	0.27459
73	0.272	0.29425	1.8488	0.046	0.12101	0.7603	0.004	0.03568	0.2242
74	0.045	0.11968	0.75199	0.044	0.11835	0.74359	0.002	0.02523	0.15853
75	9.63E-04	0.0175	0.10998	0.008	0.05046	0.31707	0.234	0.27292	1.7148
76	0.057	0.1347	0.84634	0.065	0.14384	0.90378	0.002	0.02523	0.15853
77	0.347	0.33235	2.08819	0.107	0.18455	1.15957	0.04	0.11284	0.70898
78	9.63E-04	0.0175	0.10998	9.63E-04	0.0175	0.10998	9.63E-04	0.0175	0.10998
79	0.003	0.0309	0.19416	0.123	0.19787	1.24325	0.128	0.20185	1.26826
80	9.63E-04	0.0175	0.10998	9.63E-04	0.0175	0.10998	0.009	0.05352	0.3363
81	0.276	0.2964	1.86234	9.63E-04	0.0175	0.10998	0.015	0.0691	0.43416
82	0.07	0.14927	0.93789	0.003	0.0309	0.19416	0.008	0.05046	0.31707
83	0.106	0.18369	1.15414	0.015	0.0691	0.43416	9.63E-04	0.0175	0.10998
84	9.63E-04	0.0175	0.10998	0.003	0.0309	0.19416	0.037	0.10852	0.68188
85	0.004	0.03568	0.2242	9.63E-04	0.0175	0.10998	0.011	0.05917	0.37179
86	0.003	0.0309	0.19416	0.002	0.02523	0.15853	0.025	0.08921	0.5605
87	0.046	0.12101	0.7603	9.63E-04	0.0175	0.10998	0.003	0.0309	0.19416
88	0.064	0.14273	0.8968	0.009	0.05352	0.3363	0.002	0.02523	0.15853
89	0.003	0.0309	0.19416	0.215	0.2616	1.64371	0.023	0.08556	0.53761
90	0.123	0.19787	1.24325	9.63E-04	0.0175	0.10998	0.024	0.0874	0.54917
91	9.63E-04	0.0175	0.10998	0.01	0.05642	0.35449	0.002	0.02523	0.15853
92	9.63E-04	0.0175	0.10998	0.003	0.0309	0.19416	0.008	0.05046	0.31707
93	0.005	0.03989	0.25066	0.299	0.3085	1.93839	0.06	0.1382	0.86832
94	0.002	0.02523	0.15853	0.321	0.31965	2.00843	9.63E-04	0.0175	0.10998
95	0.008	0.05046	0.31707	0.014	0.06676	0.41944	0.116	0.19216	1.20735
96	9.63E-04	0.0175	0.10998	0.045	0.11968	0.75199	0.062	0.14048	0.88267
97	0.021	0.08176	0.51371	9.63E-04	0.0175	0.10998	9.63E-04	0.0175	0.10998
98	0.113	0.18965	1.19164	0.17	0.23262	1.4616	0.015	0.0691	0.43416
99	0.028	0.09441	0.59318	0.003	0.0309	0.19416	0.048	0.12361	0.77665
100	0.021	0.08176	0.51371	0.022	0.08368	0.52579	9.63E-04	0.0175	0.10998
101	0.003	0.0309	0.19416	0.162	0.22708	1.4268	0.006	0.0437	0.27459
102	0.069	0.1482	0.93117	0.009	0.05352	0.3363	0.002	0.02523	0.15853
103	0.005	0.03989	0.25066	9.63E-04	0.0175	0.10998	9.63E-04	0.0175	0.10998
104	0.167	0.23056	1.44865	9.63E-04	0.0175	0.10998	0.022	0.08368	0.52579
105	0.012	0.0618	0.38833	0.009	0.05352	0.3363	0.038	0.10998	0.69103
106	0.003	0.0309	0.19416	0.058	0.13587	0.85373	0.003	0.0309	0.19416
107	0.015	0.0691	0.43416	0.009	0.05352	0.3363	9.63E-04	0.0175	0.10998
108	0.053	0.12989	0.8161	0.108	0.18541	1.16498	0.037	0.10852	0.68188
109	0.125	0.19947	1.25331	0.004	0.03568	0.2242	9.63E-04	0.0175	0.10998
110	9.63E-04	0.0175	0.10998	0.006	0.0437	0.27459	0.026	0.09097	0.5716
111	0.005	0.03989	0.25066	0.003	0.0309	0.19416	0.031	0.09934	0.62415
112	9.63E-04	0.0175	0.10998	0.285	0.30119	1.89246	0.003	0.0309	0.19416
113	0.012	0.0618	0.38833	0.017	0.07356	0.4622	0.064	0.14273	0.8968
114	0.07	0.14927	0.93789	0.998	0.56363	3.54136	0.005	0.03989	0.25066
115	0.035	0.10555	0.66319	0.221	0.26523	1.66648	9.63E-04	0.0175	0.10998
116	0.013	0.06433	0.40418	0.033	0.10249	0.64396	0.003	0.0309	0.19416
117	0.117	0.19298	1.21254	0.035	0.10555	0.66319	0.005	0.03989	0.25066
118	0.044	0.11835	0.74359	0.007	0.0472	0.29659	0.004	0.03568	0.2242
119	0.097	0.17572	1.10406	0.042	0.11562	0.72649	0.009	0.05352	0.3363
120	0.147	0.21631	1.35914	0.069	0.1482	0.93117	9.63E-04	0.0175	0.10998
121	0.053	0.12989	0.8161	0.017	0.07356	0.4622	0.011	0.05917	0.37179
122	9.63E-04	0.0175	0.10998	0.011	0.05917	0.37179	0.09	0.16926	1.06347
123	0.01	0.05642	0.35449	0.005	0.03989	0.25066	9.63E-04	0.0175	0.10998
124	0.064	0.14273	0.8968	0.373	0.34457	2.16501	9.63E-04	0.0175	0.10998
125	0.07	0.14927	0.93789	0.396	0.35504	2.23076	0.004	0.03568	0.2242
126	0.129	0.20264	1.27321	0.287	0.30225	1.89909	0.008	0.05046	0.31707

127	0.195	0.24914	1.56539	0.043	0.11699	0.73509	0.015	0.0691	0.43416
128	9.63E-04	0.0175	0.10998	0.009	0.05352	0.3363	9.63E-04	0.0175	0.10998
129	0.002	0.02523	0.15853	0.002	0.02523	0.15853	0.002	0.02523	0.15853
130	0.085	0.16449	1.03351	9.63E-04	0.0175	0.10998	0.003	0.0309	0.19416
131	9.63E-04	0.0175	0.10998	0.097	0.17572	1.10406	0.004	0.03568	0.2242
132	0.12	0.19544	1.22799	0.002	0.02523	0.15853	0.048	0.12361	0.77665
133	0.046	0.12101	0.7603	0.544	0.41613	2.61459	0.016	0.07136	0.4484
134	0.327	0.32263	2.02712	0.003	0.0309	0.19416	9.63E-04	0.0175	0.10998
135	0.123	0.19787	1.24325	0.013	0.06433	0.40418	0.055	0.13231	0.83135
136	0.045	0.11968	0.75199	0.054	0.13111	0.82376	0.018	0.07569	0.4756
137	0.134	0.20653	1.29765	0.106	0.18369	1.15414	0.006	0.0437	0.27459
138	0.025	0.08921	0.5605	9.63E-04	0.0175	0.10998	9.63E-04	0.0175	0.10998
139	0.012	0.0618	0.38833	0.008	0.05046	0.31707	0.111	0.18797	1.18104
140	9.63E-04	0.0175	0.10998	0.894	0.53345	3.35177	0.057	0.1347	0.84634
141	9.63E-04	0.0175	0.10998	0.002	0.02523	0.15853	0.055	0.13231	0.83135
142	0.094	0.17298	1.08685	0.002	0.02523	0.15853	0.041	0.11424	0.71779
143	0.126	0.20027	1.25832	0.051	0.12741	0.80055	9.63E-04	0.0175	0.10998
144	0.011	0.05917	0.37179	0.321	0.31965	2.00843	9.63E-04	0.0175	0.10998
145	0.08	0.15958	1.00265	0.246	0.27983	1.75822	0.019	0.07777	0.48863
146	0.17	0.23262	1.4616	9.63E-04	0.0175	0.10998	0.051	0.12741	0.80055
147	0.011	0.05917	0.37179	0.051	0.12741	0.80055	0.017	0.07356	0.4622
148	0.633	0.44888	2.82037	0.002	0.02523	0.15853	9.63E-04	0.0175	0.10998
149	0.362	0.33945	2.13284	0.067	0.14604	0.91758	9.63E-04	0.0175	0.10998
150	0.128	0.20185	1.26826	9.63E-04	0.0175	0.10998	0.008	0.05046	0.31707
151	0.522	0.40762	2.56118	0.124	0.19867	1.24829	0.119	0.19462	1.22286
152	0.006	0.0437	0.27459	0.268	0.29207	1.83515	0.04	0.11284	0.70898
153	0.019	0.07777	0.48863	0.804	0.50589	3.17858	0.005	0.03989	0.25066
154	0.094	0.17298	1.08685	0.062	0.14048	0.88267	9.63E-04	0.0175	0.10998
155	0.01	0.05642	0.35449	0.145	0.21484	1.34986	0.014	0.06676	0.41944
156	0.043	0.11699	0.73509	0.012	0.0618	0.38833	0.005	0.03989	0.25066
157	0.041	0.11424	0.71779	0.047	0.12231	0.76852	0.013	0.06433	0.40418
158	0.049	0.12489	0.7847	9.63E-04	0.0175	0.10998	0.011	0.05917	0.37179
159	0.045	0.11968	0.75199	0.043	0.11699	0.73509	0.003	0.0309	0.19416
160	0.004	0.03568	0.2242	0.036	0.10705	0.6726	0.018	0.07569	0.4756
161	0.009	0.05352	0.3363	0.213	0.26038	1.63604	0.014	0.06676	0.41944
162	9.63E-04	0.0175	0.10998	0.396	0.35504	2.23076	0.06	0.1382	0.86832
163	0.002	0.02523	0.15853	0.104	0.18195	1.1432	0.014	0.06676	0.41944
164	0.002	0.02523	0.15853	0.049	0.12489	0.7847	0.075	0.15451	0.97081
165	0.122	0.19706	1.23818	0.002	0.02523	0.15853	0.01	0.05642	0.35449
166	0.017	0.07356	0.4622	0.074	0.15348	0.96432	0.358	0.33757	2.12103
167	0.092	0.17113	1.07522	9.63E-04	0.0175	0.10998	0.031	0.09934	0.62415
168	9.63E-04	0.0175	0.10998	0.012	0.0618	0.38833	0.008	0.05046	0.31707
169	0.012	0.0618	0.38833	0.069	0.1482	0.93117	0.003	0.0309	0.19416
170	0.252	0.28322	1.77953	9.63E-04	0.0175	0.10998	9.63E-04	0.0175	0.10998
171	0.012	0.0618	0.38833	0.003	0.0309	0.19416	0.003	0.0309	0.19416
172	9.63E-04	0.0175	0.10998	9.63E-04	0.0175	0.10998	0.002	0.02523	0.15853
173	0.02	0.07979	0.50133	9.63E-04	0.0175	0.10998	9.63E-04	0.0175	0.10998
174	0.052	0.12866	0.80836	0.003	0.0309	0.19416	0.113	0.18965	1.19164
175	0.044	0.11835	0.74359	0.194	0.2485	1.56137	0.009	0.05352	0.3363
176	0.077	0.15656	0.98367	9.63E-04	0.0175	0.10998	0.011	0.05917	0.37179
177	0.069	0.1482	0.93117	0.233	0.27233	1.71113	0.02	0.07979	0.50133
178	0.064	0.14273	0.8968	0.291	0.30435	1.91228	9.63E-04	0.0175	0.10998
179	0.002	0.02523	0.15853	0.273	0.29479	1.85219	0.002	0.02523	0.15853
180	0.094	0.17298	1.08685	0.064	0.14273	0.8968	0.067	0.14604	0.91758
181	0.003	0.0309	0.19416	0.037	0.10852	0.68188	9.63E-04	0.0175	0.10998
182	0.018	0.07569	0.4756	0.007	0.0472	0.29659	0.047	0.12231	0.76852
183	0.011	0.05917	0.37179	9.63E-04	0.0175	0.10998	0.09	0.16926	1.06347
184	0.123	0.19787	1.24325	0.009	0.05352	0.3363	9.63E-04	0.0175	0.10998
185	0.002	0.02523	0.15853	0.002	0.02523	0.15853	0.003	0.0309	0.19416
186	0.011	0.05917	0.37179	0.008	0.05046	0.31707	0.003	0.0309	0.19416
187	0.055	0.13231	0.83135	0.002	0.02523	0.15853			
188	0.095	0.17389	1.09261	9.63E-04	0.0175	0.10998			
189	0.057	0.1347	0.84634	0.098	0.17662	1.10973			
190	0.105	0.18282	1.14868	0.216	0.26221	1.64752			
191	0.067	0.14604	0.91758	9.63E-04	0.0175	0.10998			
192	0.048	0.12361	0.77665	0.016	0.07136	0.4484			
193	0.071	0.15033	0.94457	0.277	0.29694	1.86571			

194	0.066	0.14494	0.9107	1.12	0.59708	3.75158			
195	0.08	0.15958	1.00265	0.038	0.10998	0.69103			
196	0.101	0.1793	1.12659	0.476	0.38925	2.44573			
197	0.055	0.13231	0.83135	0.036	0.10705	0.6726			
198	0.066	0.14494	0.9107	0.032	0.10093	0.63413			
199	0.101	0.1793	1.12659	0.037	0.10852	0.68188			
200	0.042	0.11562	0.72649	0.102	0.18019	1.13215			
201	0.073	0.15244	0.95778	0.267	0.29153	1.83173			
202	0.231	0.27116	1.70377	0.162	0.22708	1.4268			
203	0.115	0.19133	1.20214	9.63E-04	0.0175	0.10998			
204	0.544	0.41613	2.61459	0.002	0.02523	0.15853			
205	0.056	0.13351	0.83888	0.036	0.10705	0.6726			
206	0.406	0.35949	2.25875	0.039	0.11142	0.70006			
207	9.63E-04	0.0175	0.10998	0.003	0.0309	0.19416			
208	9.63E-04	0.0175	0.10998	0.108	0.18541	1.16498			
209	0.147	0.21631	1.35914	9.63E-04	0.0175	0.10998			
210	0.458	0.38182	2.39904	9.63E-04	0.0175	0.10998			
211	9.63E-04	0.0175	0.10998	9.63E-04	0.0175	0.10998			
212	9.63E-04	0.0175	0.10998	9.63E-04	0.0175	0.10998			
213	0.009	0.05352	0.3363	0.021	0.08176	0.51371			
214	0.002	0.02523	0.15853	0.042	0.11562	0.72649			
215	0.026	0.09097	0.5716	0.074	0.15348	0.96432			
216	0.085	0.16449	1.03351	9.63E-04	0.0175	0.10998			
217	0.029	0.09608	0.60368	0.002	0.02523	0.15853			
218	0.323	0.32065	2.01468	0.003	0.0309	0.19416			
219	0.493	0.39614	2.48902	0.002	0.02523	0.15853			
220	0.256	0.28546	1.7936	--	--	--			
221	0.048	0.12361	0.77665	--	--	--			
222	9.63E-04	0.0175	0.10998	--	--	--			
223	0.065	0.14384	0.90378	--	--	--			
224	0.02	0.07979	0.50133						
225	9.63E-04	0.0175	0.10998						
226	0.051	0.12741	0.80055						
227	9.63E-04	0.0175	0.10998						
228	0.04	0.11284	0.70898						
229	9.63E-04	0.0175	0.10998						
230	0.002	0.02523	0.15853						
231	0.088	0.16737	1.05159						
232	0.003	0.0309	0.19416						
233	0.011	0.05917	0.37179						
234	0.09	0.16926	1.06347						
235	0.02	0.07979	0.50133						
236	0.143	0.21335	1.34052						
237	0.013	0.06433	0.40418						
238	0.043	0.11699	0.73509						
239	0.074	0.15348	0.96432						
240	0.09	0.16926	1.06347						
241	0.108	0.18541	1.16498						
242	0.01	0.05642	0.35449						
243	0.048	0.12361	0.77665						
244	0.057	0.1347	0.84634						
245	0.003	0.0309	0.19416						
246	0.076	0.15554	0.97726						
247	0.002	0.02523	0.15853						
248	0.092	0.17113	1.07522						
249	0.064	0.14273	0.8968						
250	0.027	0.09271	0.58249						
251	0.069	0.1482	0.93117						
252	9.63E-04	0.0175	0.10998						
253	9.63E-04	0.0175	0.10998						
254	0.096	0.17481	1.09835						
255	0.251	0.28266	1.776						
256	9.63E-04	0.0175	0.10998						
257	0.05	0.12616	0.79267						
258	0.004	0.03568	0.2242						
259	0.082	0.16156	1.01511						
260	0.006	0.0437	0.27459						

261	0.002	0.02523	0.15853						
262	0.095	0.17389	1.09261						
263	0.013	0.06433	0.40418						
264	0.071	0.15033	0.94457						
265	0.006	0.0437	0.27459						
266	9.63E-04	0.0175	0.10998						
267	9.63E-04	0.0175	0.10998						
268	9.63E-04	0.0175	0.10998						
269	0.141	0.21185	1.33111						
270	0.04	0.11284	0.70898						
271	0.318	0.31815	1.99903						
272	0.073	0.15244	0.95778						
273	0.042	0.11562	0.72649						
274	0.306	0.31209	1.96095						
275	0.159	0.22497	1.41353						
276	0.003	0.0309	0.19416						
277	0.002	0.02523	0.15853						
278	0.007	0.0472	0.29659						
279	0.054	0.13111	0.82376						
280	0.121	0.19625	1.2331						
281	0.017	0.07356	0.4622						
282	0.142	0.2126	1.33582						
283	0.06	0.1382	0.86832						
284	0.002	0.02523	0.15853						
285	9.63E-04	0.0175	0.10998						
286	0.126	0.20027	1.25832						
287	9.63E-04	0.0175	0.10998						
288	0.068	0.14712	0.9244						
289	0.078	0.15757	0.99004						
290	0.09	0.16926	1.06347						
291	0.061	0.13934	0.87553						
292	0.088	0.16737	1.05159						
293	0.416	0.36389	2.2864						
294	0.391	0.35279	2.21663						
295	0.064	0.14273	0.8968						
296	0.07	0.14927	0.93789						
297	0.039	0.11142	0.70006						
298	0.068	0.14712	0.9244						
299	0.055	0.13231	0.83135						
300	0.191	0.24657	1.54925						
301	0.051	0.12741	0.80055						
302	9.63E-04	0.0175	0.10998						
303	9.63E-04	0.0175	0.10998						
304	0.051	0.12741	0.80055						
305	0.05	0.12616	0.79267						
306	0.115	0.19133	1.20214						
307	0.052	0.12866	0.80836						
308	0.061	0.13934	0.87553						
309	0.051	0.12741	0.80055						
310	9.63E-04	0.0175	0.10998						
311	9.63E-04	0.0175	0.10998						
312	0.058	0.13587	0.85373						
313	0.002	0.02523	0.15853						
314	0.064	0.14273	0.8968						
315	0.042	0.11562	0.72649						
316	0.061	0.13934	0.87553						
317	9.63E-04	0.0175	0.10998						
318	0.075	0.15451	0.97081						
319	9.63E-04	0.0175	0.10998						
320	0.003	0.0309	0.19416						
321	0.008	0.05046	0.31707						
322	9.63E-04	0.0175	0.10998						
323	0.004	0.03568	0.2242						
324	0.082	0.16156	1.01511						
325	9.63E-04	0.0175	0.10998						
326	0.004	0.03568	0.2242						
327	0.003	0.0309	0.19416						

328	9.63E-04	0.0175	0.10998						
329	0.003	0.0309	0.19416						
330	0.002	0.02523	0.15853						
331	0.04	0.11284	0.70898						
332	9.63E-04	0.0175	0.10998						
333	9.63E-04	0.0175	0.10998						
334	0.011	0.05917	0.37179						
335	0.002	0.02523	0.15853						
336	0.002	0.02523	0.15853						
337	0.056	0.13351	0.83888						
338	0.056	0.13351	0.83888						
339	0.007	0.0472	0.29659						
340	0.003	0.0309	0.19416						
341	0.004	0.03568	0.2242						
342	0.003	0.0309	0.19416						
343	0.008	0.05046	0.31707						
344	0.002	0.02523	0.15853						
345	0.006	0.0437	0.27459						
346	0.025	0.08921	0.5605						
347	9.63E-04	0.0175	0.10998						
348	0.004	0.03568	0.2242						
349	0.038	0.10998	0.69103						
350	0.016	0.07136	0.4484						
351	0.004	0.03568	0.2242						
352	0.006	0.0437	0.27459						
353	0.018	0.07569	0.4756						
354	0.035	0.10555	0.66319						
355	0.006	0.0437	0.27459						
356	0.02	0.07979	0.50133						
357	0.065	0.14384	0.90378						
358	0.015	0.0691	0.43416						
359	0.01	0.05642	0.35449						
360	0.038	0.10998	0.69103						
361	0.002	0.02523	0.15853						
362	0.003	0.0309	0.19416						
363	0.133	0.20576	1.2928						
364	9.63E-04	0.0175	0.10998						
365	0.051	0.12741	0.80055						
366	0.003	0.0309	0.19416						
367	0.043	0.11699	0.73509						
368	9.63E-04	0.0175	0.10998						
369	9.63E-04	0.0175	0.10998						
370	0.003	0.0309	0.19416						
371	0.009	0.05352	0.3363						
372	0.007	0.0472	0.29659						
373	0.004	0.03568	0.2242						

Supplementary Note 1: Electrostatic repulsions lower the oxidation potential of hydroxide anions

Semi-empirical molecular dynamics (MD) and quantum chemistry were used to study the effect of mutual electrostatic repulsion on the one electron oxidation potential of HO^- under different conditions. To approximately model 1 M, 2 M and 3 M hydroxide aqueous solution, we set up an initial cubic box using the Packmol program,[4] the box is filled with respectively 1, 2 and 3 hydroxide and 100 water molecules. The box length of the box is 14.46 angstrom based on the density of water. A 1ns NVT GFN-xTB MD simulation was then conducted. In the MD simulation, the temperature is kept in the room temperature, the coordinates of the system were dumped every 50 fs, the time step is set as 4 fs, all bonds were constrained using the SHAKE algorithm. The Travis program[5] was used to extract 100 snapshots from the MD trajectory, in each snapshot, the solute (1 HO^- or 1 $HO^•$) was placed in the centre of the simulation box and the nearest 52 water molecules were taken to simulate a 1M concentration of the solute. Similarly, for 2 M, they are respectively 2 HO^- or 1 HO^- and 1$HO^•$ in 52 water molecules. For 3 M, they are respectively 3 HO^- or 2 HO^- and 1$HO^•$ in 51 water molecules. Note here that what we are modelling is the excess concentrations of OH anion, thus no cation is included.

For each of the 100 snapshots of each MD trajectory (totally 6 trajectories and 600 snapshots), the GFN2-xTB method[6] implemented in the xtb[7] code (version 6.2.3) was used to optimize the structure and the most stable cluster was taken to further optimization and frequency calculation using B97-3c method[8] with the ORCA program.[9] The optimized gas-phase structures of OH^- and $HO^•$ system are shown respectively Supplementary Fig. 12 and the optimized coordinates are provided in the supplementary data. Gas-phase high-level single point energies are calculated using the RI-PWPB95-D3(BJ)/def2-TZVPP theoretical level with the RI approximation also in ORCA[9] program (version 4.2.1). A further ONIOM correction to the CCSD(T)/CBS level (via cc-pVTZ to QZ extrapolation) is then included based on the energy difference between this level and RI-PWPB95-D3(BJ)/def2-TZVPP level, as calculated for the one electron oxidation of HO^- in isolation. Finally, reaction energies for the one electron oxidation of HO^- were converted to oxidation potentials vs SHE using the recommended[10] value of 4.281V for the reference electrode and a value of –0.01194 kcal/mol for the energy of the electron, based on the Boltzmann statistics. Results for the 1 M, 2 M and 3 M conditions are provided in Supplementary Table 2; corresponding total energies are provided in Supplementary Table 3.

This approach uses very high levels of theory, includes a very large cluster of explicit solvent (52 waters), uses 600 snapshots to obtain the geometry of the ensemble, but does not properly account for the thermal energy and entropy of the system. As a result, the oxidation potential obtained for the 1 M system, 1.71 V vs SHE, is slightly lower than the experimental value, +1.902 ± 0.017.[2] However, because the electrostatic destabilization of the HO^-, due to the excess unbalanced ions, is expected to affect primarily the electronic energy and not the thermal energy or entropy, it is expected that this approach is able to capture properly the *effect* of electrostatic repulsion on the oxidation potential. From Supplementary Table 2, it is seen that the oxidation potential decreases from 1.71 V (1 M) to 0.77 V (2 M) to –0.55 V (3 M). This may be an overestimate of the repulsion effects, which may to be attenuated to some extent by the opposing electric field due to the bubble surface. Unfortunately, it is not practical to model the field effects and the full 52 water system at the same time. However, background field effects on the isolated reaction (see Supplementary Note 5, where data for HO^- and HO• are provided at various field strengths) are negligible, the effects determined here due to HO^- mutual repulsion are very large. Thus, even if the absolute numbers are subject to uncertainty, the trends are clear: HO^- mutual repulsion leads to significant lowering of the HO^- oxidation potential to

the extent that even at 2 M of unbalanced charge, the oxidation becomes feasible at the experimentally applied potential of 1.2 V.

Finally, we note again that all the modelling in this section corresponds to the excess OH^- conditions, so no cations are included. However, we run a test to study whether the co-existence of OH^- and H_3O^+ is possible at the experimental concentrations of *excess* HO^-. In detail, 10 HO^-, 9 H_3O^+ and 34 water molecules are placed in a cubic box with length as 11.67 angstrom. This corresponds to an excess of 1 M HO^- but in the presence of additional balanced HO^- and H_3O^+. 100 snapshots are taken from the trajectory of GFN-xTB MD simulation and optimized with GFN2-xTB method. It was found that the HO^- and H_3O^+ are recombined to water molecules in the process of geometry optimization (example snapshots see Supplementary Figure 13), which proves the co-existence of HO^- and H_3O^+ in the bubble corona seems unlikely.

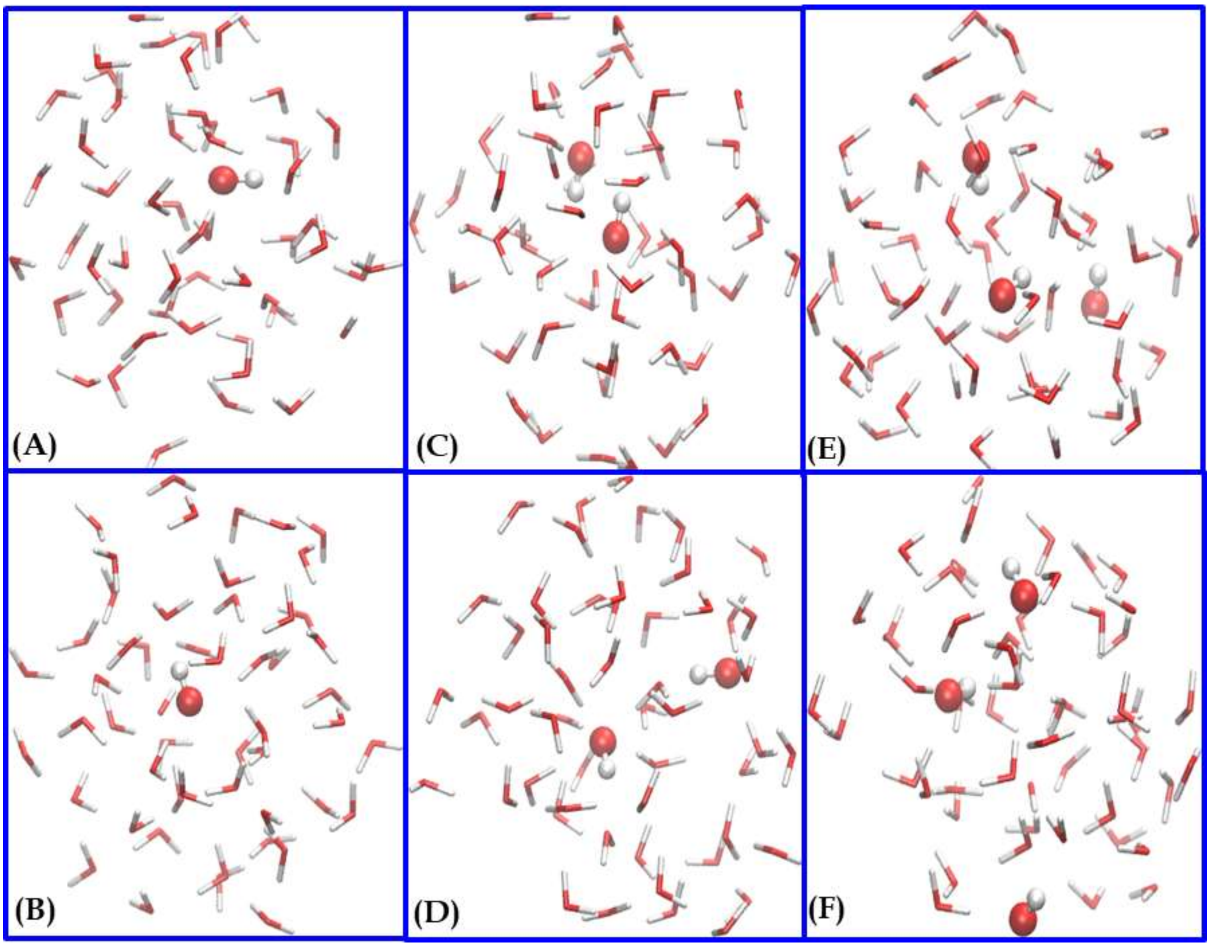

Supplementary Figure 12. Systems of OH- and $OH^•$ in 1M (A and B), 2M (C and D) and 3M (E and F) conditions. The OH- and OH* are in VDW drawing style, the water molecules are in the licorice style. The pictures are rendered using the VMD program.[11]

Supplementary Table 2. Reaction energies for 1 electron oxidation of HO^{-} (kcal mol^{-1}) and associated oxidation potentials (V vs SHE) as a function of the concentration of HO^{-} ions[a]

	1M	2M	3M
Reaction energy (kcal/mol)	138.16	116.45	86.07
Oxidation Potential (V vs SHE)	1.71	0.77	-0.55
Effect of Conc. (V)	0.00	-0.94	-2.26

[a]Energies provided here are the electronic energies of the full solute-solvent ensemble, corrected to the CCSD(T)/CBS limit using RI-PWPB95-D3(BJ)/def2-TZVPP energies with an ONIOM correction calculated using the isolated species as the core (see Supplementary Table 3).

Supplementary Table 3. All component energies (hartrees) used to obtain the oxidation potentials[a]

	$HO^{\bullet}$	HO^{-}
isolated $HO^{\bullet}$ or HO^{-} CCSD(T)/CBS	-75.67590	-75.72655
isolated $HO^{\bullet}$ or HO^{-} RI-PWPB95-D3(BJ)/def2-TZVPP	-75.72144	-75.75918
1M cluster RI-PWPB95-D3(BJ)/def2-TZVPP	-4050.27539	-4050.48265
1M cluster CCSD(T)/CBS	-4050.22985	-4050.45003
2M cluster RI-PWPB95-D3(BJ)/def2-TZVPP	-4126.16854	-4126.34120
2M cluster CCSD(T)/CBS	-4126.12300	-4126.30857
3M cluster RI-PWPB95-D3(BJ)/def2-TZVPP	-4202.02444	-4202.14868
3M cluster CCSD(T)/CBS	-4201.97890	-4202.11606

[a]Energies provided here are the electronic energies calculated using B97-3c optimized geometries.

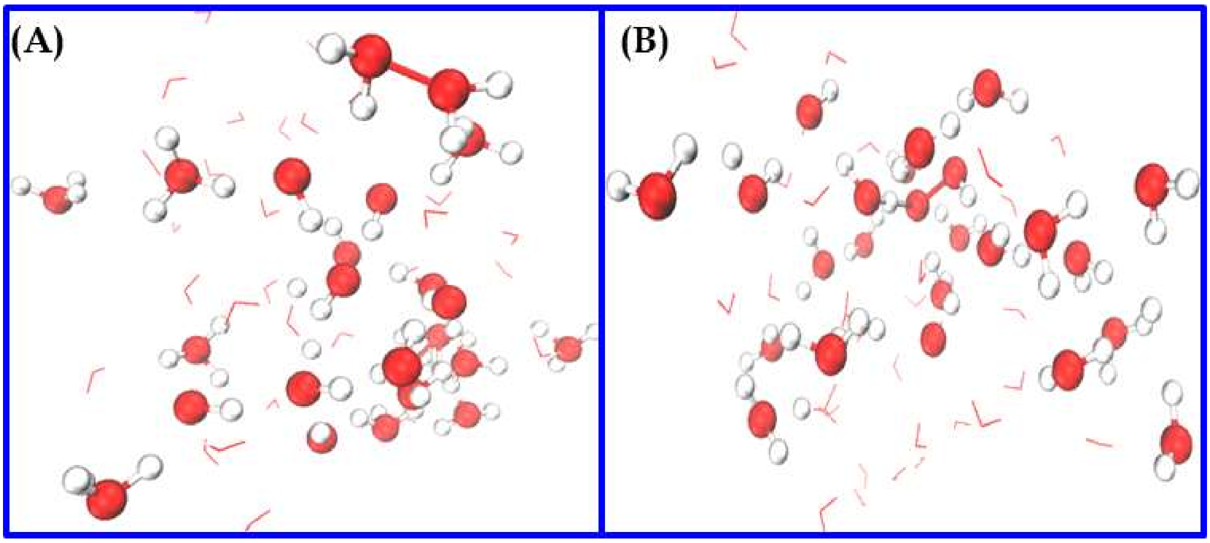

Supplementary Figure 13 Systems of OH^{-} and H_3O^{+} before (A) and after (B) geometry optimization.

Supplementary Note 2: Determination of the hydroxide ion concentration surrounding a bubble

The zeta potential (ζ) was obtained using the Smoluchowski equation[12]

$$v = \frac{\varepsilon\varepsilon^0\zeta}{\eta}E$$

Where v is the bubble velocity, ε the relative dielectric permittivity of the liquid, ε^0 the electrical permittivity in the vacuo, η the viscosity of the liquid and E the applied potential.

Loeb et al. proposed a relationship between ζ and the surface charge σ of a spherical particle[13]

$$\sigma = \frac{\varepsilon\varepsilon^0\kappa kT}{Ze}\left[2sinh\left(\frac{Ze\zeta}{2kT}\right) + \frac{4}{\kappa R}\right]tanh\left[\frac{Ze\zeta}{4kT}\right]$$

Where κ is the Debye length, k the Boltzmann constant, T the temperature, Z the number of charges (-1 for OH^-), e is the elementary charge, and R the particle radius.

Once σ is known, the surface concentration can be obtained.

Supplementary Note 3: presence of an OH^- excess at the gas–water interface, as determined by bulk pH measurements in gas/water emulsions

The experimental bulk pH drop, from pH 7.2 of the quiescent water sample, to pH 6.4 upon bubbling, corresponds to a drop in the bulk $[OH^-]$ of 4.2×10^{-11} mol L^{-1} in response to an increase of the gas–water interface. The total new interfacial area (ca. 3.1×10^3 cm^2) was estimated by means of optical microscopy (see Supplementary Video 4) counting the particles per unit of volume (9.3×10^7 particles L^{-1}) and by setting the average particle diameter to 50 µm. For experiments where the quiescent pH was increased to pH~12, the increased of bulk OH^- upon bubbling nitrogen through the water sample increased to 1.3×10^{-7} mol cm^{-2} (Supplementary Fig. 5).

Supplementary Note 4: Electrodeposited film characterization

The anodically formed polyluminol films were characterized by X-ray photoelectron spectroscopy (XPS) and cyclic voltammetry. The cyclic voltammetry of the polymer in 0.5 M of H_2SO_4 (Supplementary Fig. 14A, solid line) shows three well defined reversible redox peaks. Peaks I/IV and III/VI correspond to the transition of the three redox states of polyaniline, leucoemeraldine, emeraldine and pernigraniline, which occur at approximately 0.2 and 1.0 V, respectively.[14, 15] Peak II/V, at ca. 0.6 V, is commonly observed in polyaniline and its analogues and correlates to the presence of phanazine rings.[16] The polymer is non-conductive in basic media (Supplementary Fig. 14A, dashed line), a common feature of polyaniline films.[17]

The C1s and N1s XPS narrow scans of polyluminol samples are shown in Supplementary Fig. 14B,C. The C1s peak (Supplementary Fig. 14B) was deconvoluted into three bands: the signal at 284.3 eV, assigned to carbon atoms of the aromatic ring (C–C); the signal at 285.3 eV, assigned to anilinic nitrogen-bound carbons (C–N), and the emission at 287.4 eV, ascribed to carbons from an ester/amide group O–C=O/N–C=O. Refined areas were constrained to keep a 4:2:2 ratio (C–C: C–N: O–C=O/N–C=O). The N1s peak (Supplementary Fig. 14C) consists of two peaks: the peak at 400.4 eV attributed to the C–NH–C, and the peak at 398.9 eV corresponding to the C–N=C.[18] Areas of the refined nitrogen peaks were allowed to vary freely during the fitting, and the best fit suggests a 1:1 ratio between the two nitrogen emissions. The atomic N1s:$C1s_{287.4\,eV}$ ratio was 1:1, which suggests that what we refer to as polyluminol polymer consists probably of 50% polyaminophtalate and 50% polyluminol.

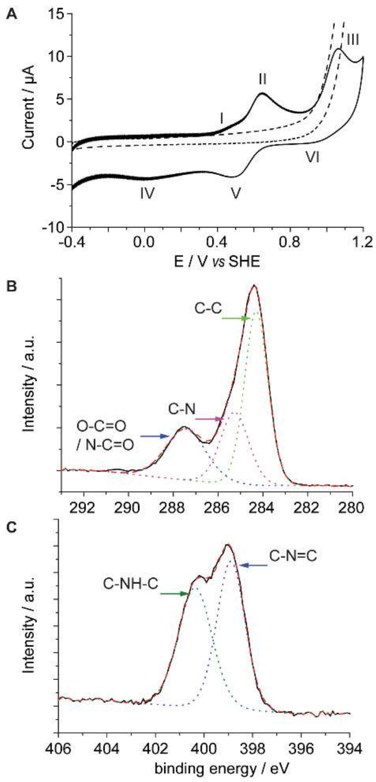

Supplementary Figure 14. Polymerization of luminol initiated by static bubbles. (A) Cyclic voltammetry (100 mV/s) of the formed polyluminol film in 0.5 M H_2SO_4 (solid line) and 0.1 M NaOH (dashed line). (B,C) Narrow XPS spectra, C 1s (B) and N 1s (C), of polyluminol films.

Supplementary Note 5: $HO^{\bullet}$ cannot form from HOO^{-} bond cleavage

In addition to considering a redox origin for the $HO^{\bullet}$ radicals, we also considered whether the electric field around a bubble was sufficient to homolytically cleave peroxide, which at the experimental pH is deprotonated, to form $HO^{\bullet}$ radicals. To this end, electronic structure calculations were performed with the Gaussian 16.C01[19] software package, at the M06-2X/6-31+G(d,p) level of theory, chosen as it has been shown previously to be sufficient for modelling electric field effects.[20, 21] The universal SMD[22] solvent model was used to capture solvent effects; water was used as the solvent environment. Optimized geometries are given below, atomic species are not supplied as no optimization is necessary. Gibbs energies were calculated via a thermocycle in which standard textbook partitions for an ideal gas under the harmonic oscillator approximation were used. Electric field effects were captured by applying the field in all three directions and using the most stabilized direction; + 2 and + 10 next to the geometry coordinates correspond to the default field specification in Gaussian.

The main possible bond cleavage pathways for HOO^{-} are shown in Supplementary Fig. 15, along with their energetics as a function of field strength. Corresponding total energies are provided in Supplementary Table 4. It is clear from the calculations that (i) the preferred pathway is (B) and so $HO^{\bullet}$ is not expected from bond cleavage; (ii) the energy for this and the other pathways is too high to expect appreciable reaction, regardless of the applied field; (iii) field has a negligible and mostly detrimental effect on the energetics, as the reactant is stabilized more than the products. This is true both an applied field of 2 atomic units (which is close to the expected experimental value) and 10 atomic units, five times higher. The formation of $HO^{\bullet}$ thus cannot be explained by bond homolysis of HOO^{-} and rather has a redox origin (Supplementary Note 1).

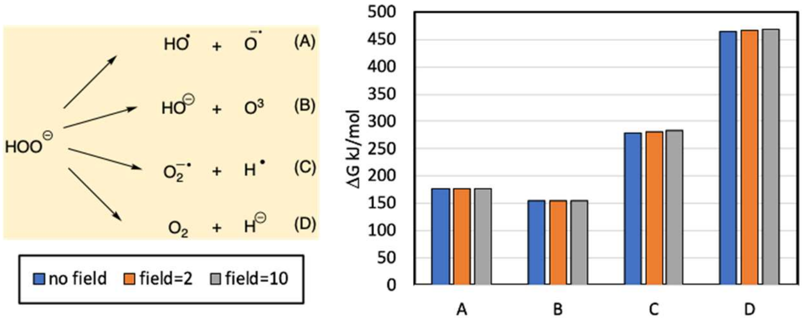

Supplementary Figure 15. Main homolytic and heterolytic bond cleavage processes. Field is given in atomic units.

Supplementary Table 4. Total energies

No Field								
Species	ZPVE kJ/mol	TC kJ/mol	S J/mol/K	E(Gas) H	E(sol) H	G(Gas) DFT kJ/mol	dE(sol) kJ/mol	G(sol) DFT kJ/mol
OOH-	35.6	10.1	225.3	-150.87806	-151.02361	-396151.8	-382.1	-396526.0
OH rad	22.6	8.7	178.2	-75.70175	-75.71145	-198776.7	-25.5	-198794.3
O rad an	0.0	6.2	149.1	-75.08551	-75.23353	-197175.2	-388.6	-197555.9
OH an	22.8	8.7	172.3	-75.75888	-75.91394	-198924.8	-407.1	-199323.9
O trip	0.0	6.2	152.5	-75.03752	-75.03928	-197050.2	-4.6	-197046.9

OO-rad	7.7	8.7	203.3	-150.27700	-150.40625	-394596.5	-339.3	-394927.9
OO neut	10.6	8.7	195.7	-150.20464	-150.20721	-394401.3	-6.7	-394400.1
H rad	0.0	6.2	114.6	-0.49668	-0.49439	-1332.0	6.0	-1318.1
H-	0.0	6.2	108.8	-0.44911	-0.62543	-1205.4	-462.9	-1660.4

Field =2 au

Species	ZPVE kJ/mol	TC kJ/mol	S J/mol/K	E(Gas) H	E(sol) H	G(Gas) DFT kJ/mol	dE(sol) kJ/mol	G(sol) DFT kJ/mol
OOH-	35.6	10.1	225.3	-150.87825	-151.02386	-396152.3	-382.3	-396526.7
OH rad	22.6	8.7	178.2	-75.70190	-75.71163	-198777.1	-25.6	-198794.8
O rad an	0.0	6.2	149.1	-75.08551	-75.23353	-197175.2	-388.6	-197555.9
OH an	22.8	8.7	172.3	-75.75903	-75.91414	-198925.1	-407.2	-199324.5
O trip	0.0	6.2	152.5	-75.03752	-75.03928	-197050.2	-4.6	-197046.9
OO-rad	7.7	8.7	203.3	-150.27701	-150.40625	-394596.5	-339.3	-394927.9
OO neut	10.6	8.7	195.7	-150.20464	-150.20721	-394401.3	-6.7	-394400.1
H rad	0.0	6.2	114.6	-0.49668	-0.49440	-1332.0	6.0	-1318.1
H-	0.0	6.2	108.8	-0.44914	-0.62545	-1205.4	-462.9	-1660.4

Field =10 au

Species	ZPVE kJ/mol	TC kJ/mol	S J/mol/K	E(Gas) H	E(sol) H	G(Gas) DFT kJ/mol	dE(sol) kJ/mol	G(sol) DFT kJ/mol
OOH-	35.6	10.1	225.3	-150.87902	-151.02490	-396154.3	-383.0	-396529.4
OH rad	22.6	8.7	178.2	-75.70250	-75.71235	-198778.7	-25.9	-198796.7
O rad an	0.0	6.2	149.1	-75.08551	-75.23354	-197175.2	-388.6	-197556.0
OH an	22.8	8.7	172.3	-75.75963	-75.91492	-198926.7	-407.7	-199326.5
O trip	0.0	6.2	152.5	-75.03752	-75.03928	-197050.2	-4.6	-197046.9
OO-rad	7.7	8.7	203.3	-150.27702	-150.40626	-394596.5	-339.3	-394927.9
OO neut	10.6	8.7	195.7	-150.20465	-150.20722	-394401.3	-6.7	-394400.2
H rad	0.0	6.2	114.6	-0.49667	-0.49439	-1332.0	6.0	-1318.1
H-	0.0	6.2	108.8	-0.44911	-0.62543	-1205.4	-462.9	-1660.4

Supplementary Note 6: Polymerization mechanism

To assess the viability of alternative possible polymerization pathways, calculations were performed with density functional theory (DFT) using the Gaussian 16 software package revision C.01. [19] Geometries optimizations and frequency calculations were performed at the M06-2X/6-31+G(d,p) level of theory with the SMD[22] continuum solvent model in water. All the geometries were verified either as local minima (possessing no imaginary frequencies) or transition states (possessing only one imaginary frequency) which were confirmed via intrinsic reaction coordinate (IRC) calculations. Entropies, thermal corrections, and zero-point vibrational energies were scaled using the recommended scaling factors.[23] For all species investigated, conformational searching was performed with the energy-directed tree search (EDTS) algorithm[24] to identify conformations with lowest Gibbs free energy. Gibbs free energies were obtained via the direct method[25] that is, entropies and thermal corrections were calculated using the ideal gas partition functions using the solution phase structures and frequencies. The quasi-harmonic oscillator (QHO) approximation was applied with a cut-off value of 100 cm^{-1}.[26]

Polymerization of luminol occurs through aniline and as such the luminescent functional group can in principle remain intact through the polymerization, leading to a luminescent polymer, or it can luminesce first resulting in a non-luminescent polymer. Moreover, the reaction cascade leading to luminescence can also be diverted to produce a third type of polymer that is also non-luminescent. These three possibilities are illustrated in Supplementary Figure 16. While in principle homopolymers of A, B or C are possible, the experiments showed that 50% of the polymer was from monomer A and the remainder B and/or C.

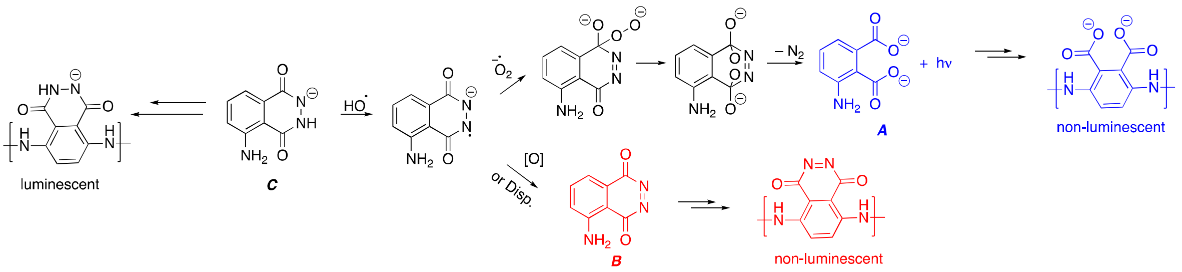

Supplementary Figure 16. Pre-polymerization.

In principle, polymerization can proceed via three different pathways, shown in Supplementary Figure 17 in blue (1-2-3), red (1-4-5-6) and green (1-4-7-8). Each begins with hydrogen atom abstraction (strictly PCET) by a hydroxyl radical from NH_2-group of the monomer (reaction 1). In the blue pathway (1-2-3), the resulting radical undergoes addition to the aromatic C=C bond at the para-position of a second monomer (reaction 2), followed by disproportionation and re-aromatisation with a further hydroxyl radical (reaction 3). This latter reaction is diffusion-limited and it is the addition reaction that determines the relative viability of this pathway. In the red (1-4-5-6) and green (1-4-7-8) pathways, diffusion limited radical-radical combination occurs instead of addition (reaction 4); however, to regenerate the active site and continue the polymerization, the product must undergo further reaction. In the red pathway, a second monomeric radical undergoes radical addition to the C=N bond of the product (reaction 5), followed by diffusion limited disproportionation and re-aromatisation. In the green pathway, propagation occurs via hydrogen abstraction from the product by a hydroxyl (reaction 6), followed by diffusion limited radical-radical coupling with another monomeric radical.

Thus, the relative viability of the 3 pathways depends on the rates of radical addition by the NH-phenyl radical to the C=C bond at the para-carbon of another monomer (2, blue pathway), versus radical addition by the C-centre of this same radical to N=C bond of the product following reaction 4 (5, red pathway), versus hydrogen atom abstraction by a hydroxyl radical from this same product (7, green pathway). This latter process is modelled as a traditional hydrogen atom transfer reaction (HAT).

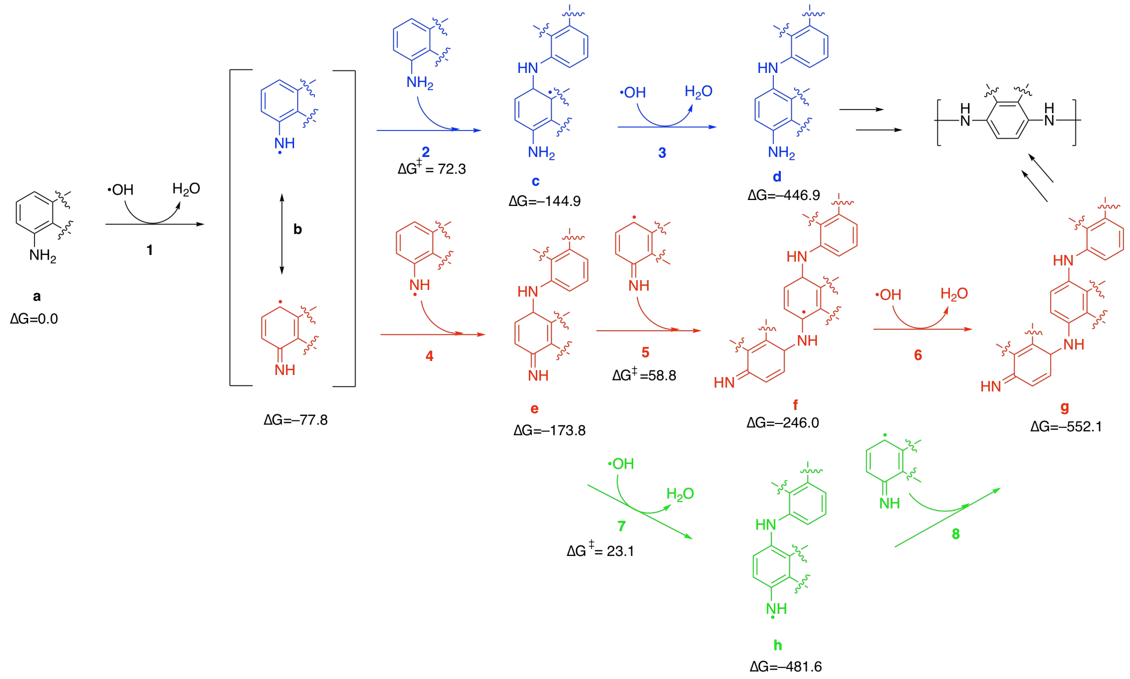

Supplementary Figure 17. Possible polymerization pathways and their energetics (298 K kJ mol^{-1}). All ΔG values are calculated with respect to the entrance channel; however, ΔG‡ values are quoted with respect to their preceding reactant or reactant complexes (whichever is lower) so as to provide a meaningful guide to the energy bottlenecks in each mechanism.

To ascertain which pathway was most favoured, we selected Monomer B and calculated the energetics of all steps in Supplementary Figure 17, including any non-diffusion-controlled barrier heights. It is clear that the thermodynamically, all pathways are consistently downhill; however, the green pathway (1-4-7-8) has a substantially lower barrier than the other two. In retrospect this is unsurprising, as this pathway involves only hydrogen atom abstractions by the reactive HO$^{\bullet}$ radical, whereas the other pathways involve radical additions by the delocalised radical of one monomer to either a C=C aromatic bond or C=N bond of another monomer unit. Moreover, the data in Supplementary Figure 17 is for the neutral monomer B; the radical additions would be expected to be even less favourable when two charged monomer units are brought together. This would be less problematic for the only reactions with a barrier in the green pathway, as these are the reaction of a monomer unit with a neutral HO$^{\bullet}$ radical. The reactions that bring two charged monomers together are instead diffusion limited radical-radical couplings, rather than slower additions, so the additional electrostatic repulsion is less likely to be problematic. For the favoured pathway, we confirmed its kinetic and thermodynamic favourability for each of the other monomers, as well as two prototypical copolymerization sequences C-A-C and C-B-C. The results are shown in Supplementary Figure 18. All polymerizations and co-polymerizations are energetically favourable. Interestingly, the homo- and

copolymerization of monomer C, the original luminol, is more favoured than the other monomers but all are energetically feasible.

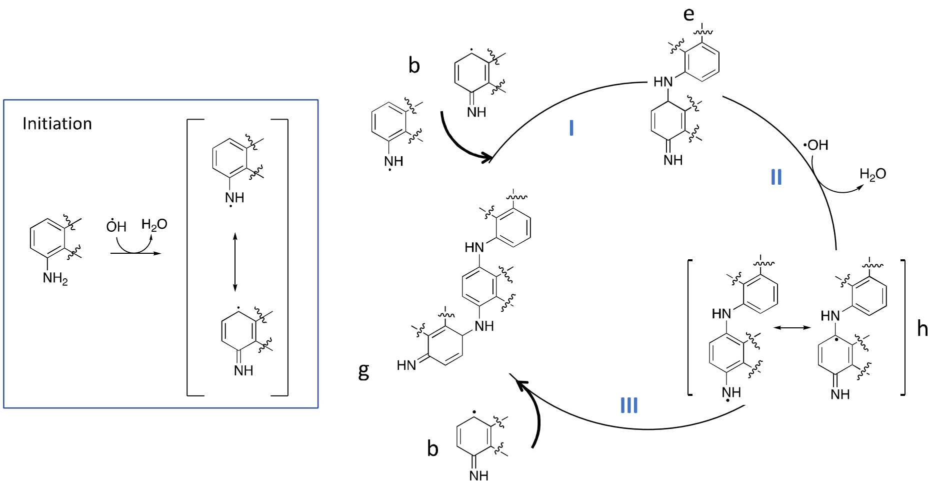

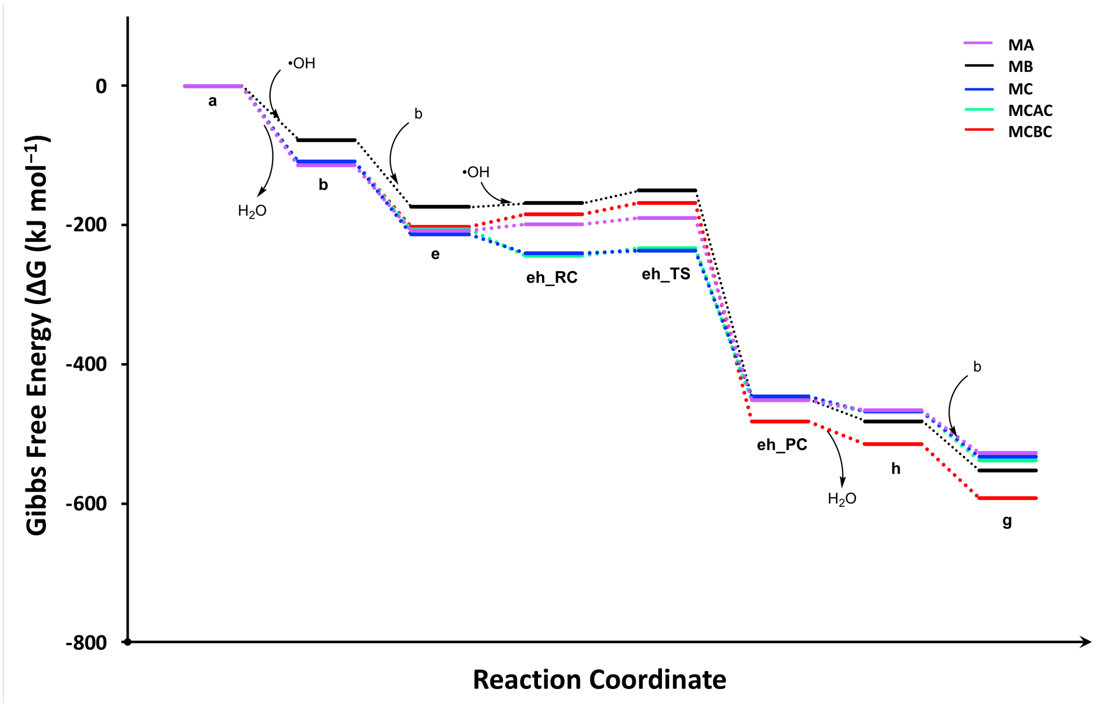

	ΔG (kJ/mol) for each species, relative to initial reactants.							ΔG‡ [a]
	b	e	eh_RC	eh_TS	eh_PC	h	g	(kJ/mol)
A	-114.2	-208.2	-198.7	-190.4	-451.1	-466.5	-528.4	17.9
B	-77.8	-173.8	-168.3	-150.7	-450.8	-481.6	-552.1	23.1
C	-107.9	-214.1	-241.1	-236.8	-447.1	-467.1	-533.7	4.3
CAC	-107.9	-206.4	-244.6	-233.9	-451.6	-468.0	-538.2	10.7

CBC	-107.9	-201.7	-184.4	-168.4	-482.5	-514.4	-592.4	33.3

Supplementary Figure 18. Reaction mechanism and the corresponding potential energy surfaces for the preferred pathway for the various monomers. All ΔG values are calculated with respect to the entrance channel; however, $\Delta G^{\ddagger}$ values are quoted with respect to their preceding reactant or reactant complexes (whichever is lower) so as to provide a meaningful guide to the energy bottlenecks in each mechanism.

While the polymerization is easily initiated by $HO^{•}$, we also considered the reaction with alternative initiating species (Supplementary Table 5). Even on the basis of thermodynamics alone, superoxide is not an effective initiator. In its protonated form, $HOO^{•}$, is more favourable; however, at pH 13, superoxide would be the predominant species. It thus seems more likely that HO• is the primary initiator and the presence of polymerization far from the bubble surface is best explained by the fact that the polymerization itself is a step process that can be restarted whenever a suitable initiator is present, even if that initiator is in low concentration. Continuous diffusion of $HO^{•}$ from the bubble surface could account for a low steady concentration of $HO^{•}$, despite its short lifetime.

Supplementary Table 5. Gibbs free energy (in kJ/mol) for the initiation step in Supplementary Figure 18 with hydroxyl ($HO^{•}$), superoxide radical ($O_2^{•-}$) and peroxyl radical ($HOO^{•}$) reacting with monomers A, B and C.

Monomer	ΔG $HO^{•}$	ΔG $O_2^{-•}$	ΔG $HOO^{•}$
A	-114.2	89.4	22.1
B	-77.8	125.8	58.6
C	-107.9	95.7	28.4

Supplementary Table 6 Raw data to calculate reaction Gibbs free energies and barriers.

	Ee	ZPVE	TC	S(J/mol^{-1}K^{-1})	H	G
OH_r	-75.711453	0.008196	0.003305	178.221000	-75.699952	-75.720190
H2O	-76.409511	0.020511	0.003782	188.764800	-76.385218	-76.406654
MA_a	-663.644722	0.117576	0.012103	433.284800	-663.515043	-663.564246
MA_b	-662.988528	0.104808	0.011771	434.439900	-662.871949	-662.921284
MA_e	-1326.045367	0.217127	0.022930	643.376900	-1325.805309	-1325.878371
MA_eh_PC	-1401.866246	0.228543	0.025507	694.554100	-1401.612196	-1401.691069
MA_eh_RC	-1401.769730	0.228241	0.025232	692.924500	-1401.516257	-1401.594945
MA_eh_TS	-1401.762716	0.223636	0.024654	680.975000	-1401.514426	-1401.591757
MA_g	-1988.488248	0.315752	0.034199	852.827800	-1988.138298	-1988.235144
MA_h	-1325.444061	0.204470	0.022800	647.330400	-1325.216791	-1325.290301
MB_a	-622.316048	0.121400	0.011222	412.857400	-622.183426	-622.230310
MB_b	-621.645875	0.108548	0.010843	413.675100	-621.526485	-621.573461
MB_bc_RC	-1243.982516	0.231817	0.022355	626.279000	-1243.728345	-1243.799464
MB_bc_TS	-1243.955740	0.232114	0.021389	613.659700	-1243.702237	-1243.771923
MB_c	-1244.017914	0.236239	0.020903	603.884300	-1243.760772	-1243.829349
MB_d	-1243.435086	0.224420	0.021156	602.452700	-1243.189510	-1243.257924
MB_e	-1243.361521	0.225423	0.021101	603.267900	-1243.114997	-1243.183504
MB_ef_RC	-1865.032646	0.336251	0.032146	806.674200	-1864.664249	-1864.755854
MB_ef_TS	-1865.010468	0.335622	0.031288	791.682400	-1864.643557	-1864.733460

MB_eh_PC	-1319.193420	0.235782	0.024127	666.337800	-1318.933511	-1319.009180
MB_eh_RC	-1319.193420	0.235782	0.024127	666.337800	-1318.933511	-1319.009180
MB_eh_TS	-1319.076566	0.232105	0.023226	648.736800	-1318.821235	-1318.894905
MB_f	-1865.066576	0.339625	0.030242	772.687200	-1864.696709	-1864.784455
MB_g	-1864.484774	0.328321	0.031167	786.323700	-1864.125286	-1864.214580
MB_h	-1242.779150	0.212650	0.020597	601.927700	-1242.545902	-1242.614257
MC_a	-623.130043	0.133101	0.011136	409.877200	-622.985806	-623.032352
MC_b	-622.471448	0.120383	0.010740	410.754600	-622.340325	-622.386970
MC_e	-1245.016582	0.248786	0.020744	593.673800	-1244.747051	-1244.814469
MC_eh_PC	-1320.831940	0.259335	0.023760	655.854700	-1320.548845	-1320.623323
MC_eh_RC	-1320.754983	0.260667	0.023472	651.901800	-1320.470845	-1320.544874
MC_eh_TS	-1320.747719	0.254754	0.023314	647.974100	-1320.469651	-1320.543235
MC_g	-1866.942448	0.363154	0.030890	776.921900	-1866.548404	-1866.636631
MC_h	-1244.412655	0.235737	0.020628	599.083600	-1244.156289	-1244.224320
MCAC_e	-1285.529701	0.232630	0.021980	622.484300	-1285.275091	-1285.345780
MCAC_eh_PC	-1361.351878	0.244331	0.024539	672.585000	-1361.083008	-1361.159387
MCAC_eh_RC	-1361.269955	0.242634	0.025317	691.393000	-1361.002004	-1361.080518
MCAC_eh_TS	-1361.263130	0.238771	0.024483	674.244900	-1360.999876	-1361.076443
MCAC_g	-1907.461171	0.347484	0.031985	801.051300	-1907.081702	-1907.172669
MCAC_h	-1284.929987	0.220153	0.021782	624.416800	-1284.688052	-1284.758961
MCBC_e	-1244.186761	0.237451	0.020874	596.342200	-1243.928436	-1243.996156
MCBC_eh_PC	-1320.020856	0.248554	0.023819	658.941600	-1319.748483	-1319.823312
MCBC_eh_RC	-1319.907639	0.248254	0.023363	649.516600	-1319.636023	-1319.709781
MCBC_eh_TS	-1319.897251	0.243466	0.022984	641.592200	-1319.630802	-1319.703661
MCBC_h	-1243.606031	0.224740	0.020536	599.348500	-1243.360755	-1243.428817
MCBC_g	-1866.139910	0.351732	0.030796	776.032500	-1865.757381	-1865.845507

Supplementary Note 7: Electrodeposition curve fitting

The electrodeposition curve (Fig. 3f of the main text) was fitted using Scharifker and Hills' theoretical treatment,[27] for a diffusion limited instantaneous nucleation growth:

$$I = ct^2 e^{-dt^3}$$

The electrodeposition curve starts with an exponential current decay that follows an electron transfer process of an adsorbed electroactive species:

$$I = ke^{-k_1 t}$$

The total current contribution can be written as:

$$I_{total} = ct^2 e^{-dt^3} + ke^{-k_1 t}$$

Where c, d, k and k_1 are constants that were allowed to move freely for the fitting (Levenberg-Marquardt algorithm). The estimated values are represented below:

	**Value**	**Standard Error**
k	909.97156	2.52786
k_1	0.02613	1.75293E-4
c	2.9462	0.03358
d	2.59851E-4	2.74049E-6

Supplementary References

1. Haapakka K. E., Kankare J. J. & Jyrki A L. Determination of the second acidity constant of luminol. *Anal. Chim. Acta* **139**, 379-382 (1982).

2. Armstrong D. A.*, et al.* Standard electrode potentials involving radicals in aqueous solution: inorganic radicals (IUPAC Technical Report). *Pure Appl. Chem.* **87**, 1139-1150 (2015).

3. Lind J., Merenyi G. & Eriksen T. E. Chemiluminescence mechanism of cyclic hydrazides such as luminol in aqueous solutions. *J. Am. Chem. Soc.* **105**, 7655-7661 (1983).

4. Martínez L., Andrade R., Birgin E. G. & Martínez J. M. PACKMOL: A package for building initial configurations for molecular dynamics simulations. *J. Comput. Chem.* **30**, 2157-2164 (2009).

5. Brehm M. & Kirchner B. TRAVIS - A Free Analyzer and Visualizer for Monte Carlo and Molecular Dynamics Trajectories. *J. Chem. Inf. Model.* **51**, 2007-2023 (2011).

6. Bannwarth C., Ehlert S. & Grimme S. GFN2-xTB—An Accurate and Broadly Parametrized Self-Consistent Tight-Binding Quantum Chemical Method with Multipole Electrostatics and Density-Dependent Dispersion Contributions. *J. Chem. Theory Comput.* **15**, 1652-1671 (2019).

7. xtb, https://github.com/grimme-lab/xtb (accessed 11 June 2020).

8. Brandenburg J. G., Bannwarth C., Hansen A. & Grimme S. B97-3c: A revised low-cost variant of the B97-D density functional method. *J. Chem. Phys.* **148**, 064104 (2018).

9. Neese F. The ORCA program system. *Wiley Interdiscip. Rev.: Comput. Mol. Sci.* **2**, 73-78 (2012).

10. Kelly C. P., Cramer C. J. & Truhlar D. G. Single-ion solvation free energies and the normal hydrogen electrode potential in methanol, acetonitrile, and dimethyl sulfoxide. *J. Phys. Chem. B* **111**, 408-422 (2007).

11. Humphrey W., Dalke A. & Schulten K. VMD: visual molecular dynamics. *J Mol Graph* **14**, 33-8, 27-8 (1996).

12. Smoluchowski M. v. Handbuch der Elektrizität und des Magnetismus. *Band II, Barth-Verlag, Leipzig*, 366-427 (1921).

13. Loeb A., Wiersema P. & Overbeek J. T. G. The Electric Double Layer Around a Spherical Colloidal Particle.). MIT Press, Cambridge (Mass.) (1961).

14. Pruneanu S., Veress E., Marian I. & Oniciu L. Characterization of polyaniline by cyclic voltammetry and UV-Vis absorption spectroscopy. *Journal of Materials Science* **34**, 2733-2739 (1999).

15. Genies E. M., Lapkowski M., Santier C. & Vieil E. Polyaniline, spectroelectrochemistry, display and battery. *Synth. Met.* **18**, 631-636 (1987).

16. Geniès E. M., Lapkowski M. & Penneau J. F. Cyclic voltammetry of polyaniline: interpretation of the middle peak. *Journal of Electroanalytical Chemistry and Interfacial Electrochemistry* **249**, 97-107 (1988).

17. Focke W. W., Wnek G. E. & Wei Y. Influence of oxidation state, pH, and counterion on the conductivity of polyaniline. *J. Phys. Chem.* **91**, 5813-5818 (1987).

18. Kumar S. N., Gaillard F., Bouyssoux G. & Sartre A. High-resolution XPS studies of electrochemically synthesized conducting polyaniline films. *Synth. Met.* **36**, 111-127 (1990).

19. Frisch M. J. T., G. W.; Schlegel, H. B.; Scuseria, G. E.; Robb, M. A.; Cheeseman, J. R.; Scalmani, G.; Barone, V.; Petersson, G. A.; Nakatsuji, H.; Li, X.; Caricato, M.; Marenich, A. V.; Bloino, J.; Janesko, B. G.; Gomperts, R.; Mennucci, B.; Hratchian, H. P.; Ortiz, J. V.; Izmaylov, A. F.; Sonnenberg, J. L.; Williams; Ding, F.; Lipparini, F.; Egidi, F.; Goings, J.; Peng, B.; Petrone, A.; Henderson, T.; Ranasinghe, D.; Zakrzewski, V. G.; Gao, J.; Rega, N.; Zheng, G.; Liang, W.; Hada, M.; Ehara, M.; Toyota, K.; Fukuda, R.; Hasegawa, J.; Ishida, M.; Nakajima, T.; Honda, Y.; Kitao, O.; Nakai, H.; Vreven, T.; Throssell, K.; Montgomery Jr., J. A.; Peralta, J. E.; Ogliaro, F.; Bearpark, M. J.; Heyd, J. J.; Brothers, E. N.; Kudin, K. N.; Staroverov, V. N.; Keith, T. A.; Kobayashi, R.; Normand, J.; Raghavachari, K.; Rendell, A. P.; Burant, J. C.; Iyengar, S. S.; Tomasi, J.; Cossi, M.; Millam, J. M.; Klene, M.; Adamo, C.; Cammi, R.; Ochterski, J. W.; Martin, R. L.; Morokuma, K.; Farkas, O.; Foresman, J. B.; Fox, D. J. Gaussian 16 Rev. C.01. Wallingford, CT (2016).

20. Aragones A. C.*, et al.* Electrostatic catalysis of a Diels-Alder reaction. *Nature* **531**, 88-91 (2016).

21. Gryn'ova G., Marshall D. L., Blanksby S. J. & Coote M. L. Switching radical stability by pH-induced orbital conversion. *Nat. Chem.* **5**, 474 (2013).

22. Marenich A. V., Cramer C. J. & Truhlar D. G. Universal solvation model based on solute electron density and on a continuum model of the solvent defined by the bulk dielectric constant and atomic surface tensions. *J. Phys. Chem. B* **113**, 6378-6396 (2009).

23. Alecu I. M., Zheng J., Zhao Y. & Truhlar D. G. Computational Thermochemistry: Scale Factor Databases and Scale Factors for Vibrational Frequencies Obtained from Electronic Model Chemistries. *Journal of Chemical Theory and Computation* **6**, 2872-2887 (2010).

24. Izgorodina E. I., Yeh Lin C. & Coote M. L. Energy-directed tree search: an efficient systematic algorithm for finding the lowest energy conformation of molecules. *Phys. Chem. Chem. Phys.* **9**, 2507-2516 (2007).

25. Ribeiro R. F., Marenich A. V., Cramer C. J. & Truhlar D. G. Use of solution-phase vibrational frequencies in continuum models for the free energy of solvation. *J. Phys. Chem. B* **115**, 14556-14562 (2011).

26. Averkiev B. B. & Truhlar D. G. Free energy of reaction by density functional theory: oxidative addition of ammonia by an iridium complex with PCP pincer ligands. *Catalysis Science & Technology* **1**, 1526-1529 (2011).

27. Scharifker B. & Hills G. Theoretical and experimental studies of multiple nucleation. *Electrochim. Acta* **28**, 879-889 (1983).
