# Supplementary material for: The corona of a surface bubble promotes electrochemical reactions
Source: Nat Commun. 2020 Dec 10;11:6323. doi: 10.1038/s41467-020-20186-0 (PMC7729901; doi:10.1038/s41467-020-20186-0)
Supplement: Supplementary file 2 — Description of Additional Supplementary Files [file 41467_2020_20186_MOESM2_ESM.pdf]

Description of Additional Supplementary Files

File Name: Supplementary Movie 1.

Description: Epifluorescence microscopy data for the detection of hydroxyl radicals in the corona of an argon bubble adhering on an ITO electrode. The electrode is immersed in an aqueous solution of sodium hydroxide (0.1 M) and 3′-(p-hydroxyphenyl) fluorescein (10 µM), and at ~17 s the electrode potential is stepped from open circuit to +1.2 V *vs* SHE.

File Name: Supplementary Movie 2.

Description: Bright field microscopy tracking the position of oxygen bubbles that are accelerated in ultrapure water towards a platinum anode under an electric field of ca. 40 V cm^{-1}.

File Name: Supplementary Movie 3.

Description: Bright field microscopy tracking the position of nitrogen bubbles that are accelerated in ultrapure water towards a platinum anode under an electric field of ca. 40 V cm^{-1}.

File Name: Supplementary Movie 4.

Description: Bright field microscopy of the gas/water emulsions with a high quantity of bubbles (9.3×10^7 particles L^{-1}) in an aqueous solution of sodium chloride (0.1 M).

File Name: Supplementary Movie 5.

Description: Chemiluminescence microscopy (10×, em 440 nm) following the anisotropic luminol polymerization reaction initiated around oxygen bubbles adhering on an ITO anode (+1.2 V *vs* SHE), in an aqueous solution of sodium hydroxide (0.1 M), luminol (0.05 M) and hydrogen peroxide (0.3 % v/v).

File Name: Supplementary Movie 6.

Description: Wide field chemiluminescence microscopy showing the anisotropic polymerization reaction initiated at the gas–liquid interface of oxygen bubbles adhering on an ITO anode (+1.2 V *vs* SHE) in an aqueous solution of sodium hydroxide (0.1 M), luminol (0.05 M) and hydrogen peroxide (0.3 % v/v).

File Name: Supplementary Movie 7.

Description: Epifluorescence microscopy for the detection of reactive oxygen species around oxygen bubbles adhering onto an ITO electrode (OCP and +1.2 V *vs* SHE) in a solution containing $DCFH_2$-DA (100 µM), sodium hydroxide (0.1 M) and hydrogen peroxide (0.3% v/v).

File Name: Supplementary Movie 8.

Description: Epifluorescence microscopy for argon bubbles deposited on an ITO electrode in a deaerated aqueous solution containing 0.1 M phosphate buffer at pH 7, 100 µM $DCFH_2$-DA and 0.3% H_2O_2. At ~15 s the potential of the electrode is stepped from OCP to +1.2 V *vs* SHE.
